# Developing Actively Targeted Nanoparticles to Fight Cancer: Focus on Italian Research

**DOI:** 10.3390/pharmaceutics13101538

**Published:** 2021-09-22

**Authors:** Monica Argenziano, Silvia Arpicco, Paola Brusa, Roberta Cavalli, Daniela Chirio, Franco Dosio, Marina Gallarate, Elena Peira, Barbara Stella, Elena Ugazio

**Affiliations:** Department of Drug Science and Technology, University of Turin, 10125 Turin, Italy; monica.argenziano@unito.it (M.A.); paola.brusa@unito.it (P.B.); roberta.cavalli@unito.it (R.C.); daniela.chirio@unito.it (D.C.); franco.dosio@unito.it (F.D.); elena.peira@unito.it (E.P.); barbara.stella@unito.it (B.S.); elena.ugazio@unito.it (E.U.)

**Keywords:** cancer therapy, active targeting, ligand, receptor, liposomes, solid lipid nanoparticles, polymeric nanocarriers, nanobubbles

## Abstract

Active targeting is a valuable and promising approach with which to enhance the therapeutic efficacy of nanodelivery systems, and the development of tumor-targeted nanoparticles has therefore attracted much research attention. In this field, the research carried out in Italian Pharmaceutical Technology academic groups has been focused on the development of actively targeted nanosystems using a multidisciplinary approach. To highlight these efforts, this review reports a thorough description of the last 10 years of Italian research results on the development of actively targeted nanoparticles to direct drugs towards different receptors that are overexpressed on cancer cells or in the tumor microenvironment. In particular, the review discusses polymeric nanocarriers, liposomes, lipoplexes, niosomes, solid lipid nanoparticles, squalene nanoassemblies and nanobubbles. For each nanocarrier, the main ligands, conjugation strategies and target receptors are described. The literature indicates that polymeric nanoparticles and liposomes stand out as key tools for improving specific drug delivery to the site of action. In addition, solid lipid nanoparticles, squalene nanoparticles and nanobubbles have also been successfully proposed. Taken together, these strategies all offer many platforms for the design of nanocarriers that are suitable for future clinical translation.

## 1. Introduction

Cancer is a leading cause of death worldwide and has a significant impact on society in terms of productivity loss and poor health-related quality of life. Current strategies for cancer treatment are mostly based on conventional chemotherapy, which is highly non-specific in targeting drugs to the cancer and can therefore affect both cancerous and normal cells [1]. To overcome these drawbacks, scientists have made intensive efforts for several decades to maximize the killing effect of chemotherapeutic agents on cancer cells and minimize the adverse effects on healthy tissues by achieving better therapy targeting/localization [2].

The application of nanomedicine in cancer therapy, with the aim of striking and selectively destroying the targets, has aroused significant interest both in the academic and the industrial fields [3,4]. Cancer nanomedicine is a constantly progressing science that is based on innovative physicochemical concepts and materials, and has been exploited to design nanotherapeutics, i.e., submicron novel drug-carrier systems, defined as nanocarriers, that may be able to overcome the many limitations of conventional drugs, such as poor water solubility, unfair oral bioavailability, non-specific biodistribution and a lack of targeting ability [5,6,7,8]. Nanomedicine has gained considerable interest due to its ability to overcome the challenges of tumor therapy, such as metastasis and multi-drug resistant or recurrent tumors [9].

Moreover, most biopharmaceutical molecules, such as nucleic acids and proteins, have poor cell-membrane permeability and are easily degraded in vivo, and this obviously prevents their direct use. Consequently, the clinical use of this kind of product in therapy should be improved with vehicles that can protect them from degradation and ensure selective release to the target [2]. The recently developed mRNA-based vaccines to prevent the SARS-CoV-2 infection, vaccines which are composed of lipid nanoparticles (NPs), are an important recent therapeutic application of a nanomedicine for nucleic-acid delivery.

Nanocarriers can encapsulate and/or entrap hydrophilic and hydrophobic drugs, modulate their release and distribution, improve drug mucosal and cell absorption and protect them from degradation [10]. Nanoparticles (NP)-based drug delivery systems (DDS) provide several advantages, as, owing to their extremely small size, they can easily accumulate into rapidly growing tumor masses. Their specific cell uptake and trafficking mechanisms allow active substances to be delivered to the target site in sufficient concentrations, thus decreasing the amounts that accumulate in undesired tissues/organs [11].

It is well known that the delivery of nanocarriers to tumors can be achieved via two targeting strategies, passive and active targeting [12]. The first allows nanocarriers to accumulate in the tumor tissue via the enhanced permeability and retention (EPR) effect [5], which also depends on the physical properties (i.e., size, conformation) and surface characteristics of the nanocarriers that, consequently, must be properly designed. Furthermore, passive targeting is limited because the EPR phenomenon in tumoral tissue is conditioned by the heterogeneity between tumor types, low or absent tumor uptake and/or retention and the differing permeability of vessels throughout a single tumor. Moreover, high interstitial pressure and the dense stroma structure often limit the homogeneous intratumoral distribution of nanocarriers, compromising the therapeutic effect [13].

The second strategy, active targeting, aims to overcome these limitations by modifying nanocarriers so that they actively bind to specific tumor cells or microenvironments. It mainly consists of the delivery of NPs to the tumor site thanks to target-site recognition by ligand molecules on the surface and an interaction with a highly expressed specific cell-surface receptor [14].

This is achieved by decorating the nanocarrier surface with ligands that bind to receptors that are overexpressed on tumor cells, thus improving the affinity for the cancer-cell surface and/or the cancer microenvironment, and enhancing drug penetration and accumulation in the tumor region. Moreover, NP platforms possess potential multifunctional interactions with both ligand and drug molecules, the latter of which are able to interact with NPs either chemically, via electrostatic and/or hydrophobic interactions, or physically, being dissolved or entrapped in the NP matrix. Ligands are highly specific for target cells/tissues. Moreover, several molecules can be attached to the nanocarrier simultaneously to increase binding to the target. The first evidence of this phenomenon was proposed in 1980 when antibodies were grafted onto the surface of liposomes [15]. Although ligands of different types have been proposed and used over time to target nanocarriers actively, an optimal targeting strategy has not yet been identified, as each has its advantages and disadvantages. The search for other molecular targets will improve delivery to the tumor area by reducing toxicity to normal tissues [16]. Perhaps, a combination of strategies may, in the future, be exploited to improve the accuracy of drug delivery, paving the way for more effective personalized therapy [17].

It should be noted that the performance of targeted anticancer nanomedicine significantly depends on the properties of the vehicles (size, surface charge, stability, degradability, safety, etc.), the ligands (nature, chemical binding, density, availability, etc.), the therapeutic agents (type, target, loading and efficiency, release, etc.) and other indications (tumor category, volume, stage, receptor density, heterogeneity, accessibility, etc.), with all of the factors intimately interacting with each other [2].

In various studies, both in vitro and in vivo, it has been shown that nanocarriers that are intended for active targeting achieve the selectivity of uptake by tumor cells through endocytosis and/or receptor-mediated cytotoxicity of traditional drugs or other active compounds such as nucleic acids and products of plant origin [18]. However, some of the disadvantages of actively targeted nanocarriers should also be mentioned. Firstly, it must be noted that their clinical use is limited to some types of cancer that express specific receptors on their cell surfaces. Furthermore, the production of targeted nanocarriers is expensive and requires appropriate technology [18].

Despite the considerable interest in this research area, and many successful nonclinical trials, only a few passively targeted nanocarriers have been approved by the regulatory agencies for cancer treatment, and no actively targeted nanocarriers have achieved this recognition [11,12].

Anyway, several nanosystems are undergoing clinical trials [19] and have shown promise for drug delivery to a variety of tumors. Most are either liposomal or polymeric, while other typologies of nanocarriers are likely to enter clinical trials in the near future, as many materials have already been deeply investigated in non-clinical tests.

Despite the exposed criticisms, there is no doubt that active receptor-based targeting has the potential to be the first-choice release strategy, and interest in the development of this kind of system has therefore significantly increased, both in terms of diagnostic applications and therapies. It is important to underline that the synergy between active and passive targeting is a good solution with which to promote nanocarrier accumulation to a desired site, particularly a tumor. The active cancer-targeting approach, being complementary to the EPR effect, is a promising tool that can improve the specific localization of both drug and carrier, and thus avoid healthy tissues and minimize off-target payloads by exploiting nanocarrier engineering [20,21]. This strategy can significantly increase the amount of a drug that is delivered to a target, compared to free drugs or passive targeting. It thereby enhances therapeutic efficacy and limits adverse effects.

Many research groups worldwide are working on actively targeted nanocarriers, and several of them are located in Italy, and include researchers from a range of disciplines, including chemists, physicists, engineers and pharmacists. The aim of the present review paper is to highlight the results of the last group, discussing the most representative examples of Italian research carried out by many Pharmaceutical Technology academic groups on the development of actively targeted NPs for cancer therapy purposes over the last 10 years. We decided to restrict the framework of this paper to Italian academic research, well aware that many non-academic research centers deal with this field, as we would prefer to highlight the strengths of Italian academic research and the role it fulfills in the national and international scenes.

A second, certainly no less important, aim of the present review paper is to underline how the advanced progress in nanocarrier-based active targeting to fight cancer is strictly related to interdisciplinary approaches and integrative dynamics in different research fields, involving collaborations between numerous Italian technology groups and EU and/or extra EU groups.

After a section describing some of the main active-targeting strategies, which will provide some context, we decided to structure the review around two main topics: polymer-based and lipid-based nanocarriers. Polymeric NPs and polymer-shelled nanobubbles are included in the former, while the latter comprises liposomes, niosomes, lipoplexes, solid lipid nanoparticles (SLNs) and squalene (SQ) NPs. Inorganic NPs, NPs for diagnostics and imaging, drug-nanocolloids, polyelectrolyte nanocapsules and stimuli-responsive NPs are not within the scope of this review, although they play an important role in cancer nanomedicine.

## 2. Ligands for Active Targeting and Receptor-Based Active Targeting Strategies

The presence of several physiological barriers and the heterogeneity of the cancer-cell microenvironment render the active-targeting approach more complex than the EPR-based one. Specific homing, enhanced retention at the target site and uptake by the target cells are the main aims of active targeting. The search for suitable anticancer targets is on-going because of the heterogeneous and adaptive nature of most tumor types [22].

A receptor can be exploited in ligand-target therapy only if it is present at a high density on pathological cells, but almost absent or inaccessible on normal ones. Moreover, it must have high specificity for the ligand and should have the ability to be internalized and recycled back to the cell surface for another round of ligand binding and the endocytosis process.

Ligands can easily attach to the receptors expressed on cancer cells and can mediate nanocarrier delivery and accumulation inside the tumor site, mainly via receptor-mediated endocytosis. Then, the drug can be released into the site for the therapeutic effect; the delivery capacity of nanocarriers is directly related to their structure and composition [14].

A broad range of ligands are used to decorate the nanocarrier surface, and these belong to the families of small molecules, carbohydrates, peptides, proteins and growth factors; ligands can be roughly classified as peptide-protein ligands and non-peptide ligands.

### 2.1. Peptide-Protein Ligands

Antibodies (Ab) are 150 kDa large Y-shaped multi-chain proteins and are the most commonly used peptide-ligands for decorating nanocarriers. Thanks to the differentiation of light and heavy chains by the variable sequences present at the tips of the protein, infinite specific and selective sites can be created to target an antigen/receptor that is overexpressed on tumor surfaces efficiently [23]. As their large size results in low tumor-cell penetration and quick elimination by the mononuclear phagocytic system (MPS) via non-specifically binding, fragments of Ab have also often been proposed for enhanced specificity against antigens or receptors that are overexpressed on tumor surfaces [24]. Various Ab and Ab fragments include the epithelial growth factor receptor (EGFR) antibody and monoclonal antibodies (mAb), raised against CD20, CD47 and Fas, to decorate the nanocarrier surface.

Peptides that are used as ligands offer many advantages in cellular targeting thanks to their small size, precise chemical structure, biocompatibility and stability [25]. Moreover, they are easy and cost-effective to produce in large amounts with high purity, and their proteolytic degradation can be prevented by chemical modification.

Transferrin (Tf) is a glycoprotein that regulates iron levels in bodily fluids by binding and sequestering it. Tf-loaded iron is internalized into cells by Tf receptor (TfR)-mediated endocytosis; lactoferrin (Lf) is a mammalian cationic iron-binding glycoprotein that belongs to the Tf family [26].

### 2.2. Non-Peptide Ligands

Molecules that are non-peptides are grouped together in this category; the most commonly used to decorate nanocarriers to target cancer cells specifically are aptamers, folic acid (FA), carbohydrates and polysaccharides. Non-peptide aptamers are short oligonucleotide sequences of either sRNA or DNA that are synthesized in vitro and that can interact with a variety of targets with high affinity and specificity. FA (vitamin B9) is a small, safe, non-immunogenic molecule that is essential for vital cellular activities such as DNA synthesis, cell division, growth and cell survival, especially in highly dividing cells or cancers. It can rapidly penetrate solid tumors, and it is easily available to form chemical conjugations with other molecules [17].

Galactose has an affinity for asialoglycoprotein receptor (ASGPR), which is overexpressed on the surface of liver cancer, meaning that it is used as a ligand.

Hyaluronic acid (HA), a naturally occurring biodegradable, biocompatible, negatively charged glycosaminoglycan, plays a crucial role in cell adhesion, migration, invasion, proliferation, differentiation and angiogenesis by binding to specific cell receptors, mainly glycoprotein CD44. It is available in a wide range of molecular weights, and its conjugation to nanocarriers has been widely used to develop HA-based targeted therapy and translate engineered nanocarriers into therapy.

In accordance with bisphosphonate-based bone-targeted therapy, alendronate and other bisphosphonates have been described as targeting agents for synergically targeting bone tumors and metastases [27,28].

After describing the most frequently used ligands for NP active targeting, a short mention must be given to a few receptor-based active-targeting strategies that must certainly be mentioned alongside the description of most important receptors.

### 2.3. Human Epidermal Growth Factor Receptor (HER)

This is a broad family that includes four components, EGFR (HER1), HER2, HER3 and HER4, and is assigned to receptor tyrosine kinase (RTK) type I. It has an important role in the control of key signaling pathways that are related to cancer-cell growth, proliferation, migration and survival. According to the assessment that EGFR is often overexpressed in epithelial tumors, such as in squamous cancer of the head and neck region [29,30,31,32], as well as in pancreatic, renal, colon, brain, ovarian and breast cancers, EGFR was the first receptor to be proposed as an interesting therapeutic target for cancer therapy, and, therefore, several promising nanoplatforms, which are based on the use of inhibitors of these factors (i.e., tyrosine kinase inhibitors), ranging from small molecules to even monoclonal antibodies, have recently been developed [15].

### 2.4. CD44 Receptor (CD44)

The CD44 receptor is a cell-surface adhesion non-kinase transmembrane receptor, which mainly binds the ubiquitous component of the extracellular membrane hyaluronan or HA with high affinity [33]. It has also been recognized to bind to several other ligands including osteopontin, collagens and matrix metalloproteinases. It is overexpressed in several cancer cells, such as lung, breast, colon, prostate, head and neck [34], and regulates metastasis via the recruitment of CD44 to the cell surface. HA is considered as the main ligand for CD44 and can bind ubiquitously expressed CD44 isoforms.

### 2.5. Folate Receptor (FL)

FL is present in humans as four isoforms (FRα, Frβ, FRγ and FRδ) and is a cysteine-rich cell-surface glycoprotein receptor. It is overexpressed in cervical, epithelial, breast, lung, ovarian, kidney, colorectal and brain tumors. FRα provides the potential for active targeting strategies, allowing both tumor localization and the specific delivery of therapeutic agents to the malignant tissue. FR is capable of binding to FA, and several FA-conjugated nanocarriers have therefore been developed. The selective uptake of FA-surface modified drug-loaded nanocarriers occurs through FR, and the cell-surface receptor–ligand complex is transported into the cell via receptor-mediated endocytosis for ligand release [35,36].

### 2.6. Prostate-Specific Membrane Antigen (PSMA)

PSMA is a type II transmembrane glycoprotein with folate hydrolase activity that is overexpressed in prostate cancer cells and, therefore, is an excellent target for imaging and therapy. It has been largely investigated as a potential antigen for specific direct vascular tumor targeting and exploited to engineer drug-loaded NPs with a PSMA ligand [37]. Although it has been recognized as a highly promising target, mainly for diagnostic applications [38], several authors have recently developed highly specific targeting agents for PSMA, which enable target-specific drug delivery to be performed [39].

### 2.7. Transferrin Receptors (TF)

The iron-free form of Tf, apotransferrin, forms Tf upon binding to Fe^3+^, then binds to Tf receptors, TfRs, that are located on the cell surface, and then it enters the cell [40]. It has been shown that TfRs are overexpressed, 2–10-fold, in most tumor cells, including breast, lung, brain, liver, ovarian prostate and colon cancer cells, and are directly involved in the process of iron homeostasis and cell proliferation, making them attractive and effective targets for site-specific antitumor drug delivery [41]. TfRs are therefore interesting targets in the development of ligand-based nanotherapies that involve the natural ligand Tf, mAbs or single-chain variable fragments (scFv) [42,43]. The Tf approach has been most thoroughly investigated for drug delivery to the brain, as the Tf ligand facilitates transcytosis of the conjugated drug-carrier systems across the blood brain barrier (BBB). TfR targeting has also been successfully exploited in ovarian carcinoma [44] and used to overcome multidrug resistance (MDR) in breast cancer [45].

### 2.8. Biotin-Specific Receptors

Biotin is an essential micronutrient that can be exploited to provide tumor-targeting properties to nanocarriers, thanks to the overexpression of biotin-specific uptake systems [46], which have recently been found to be enhanced in many cancer cells lines, including ovarian, leukemia, mastocytoma, colon, renal and breast. For these reasons, many examples of biotin being used as a targeting moiety in tumor therapy, both for therapeutic and diagnostic/theranostic purposes, have been reported in the literature [47].

### 2.9. Interleukin Receptor

The therapy of malignant gliomas, due to their complexity and the existence of the BBB, which hinders the accumulation and uptake of endogenous substances in tumors, remains a challenge despite the recent improvements. The advances in targeting technology and breakthroughs in the overexpressed receptor derive from the discovery of interleukin receptors, such as IL-13RA2, in gliomas. The discovery of such a receptor creates a benchmark in drug delivery and targeting with encouraging output in clinical investigations [48]. The discovery of interleukin-receptor overexpression on glial tumors drove the use of an interleukin 6 receptor binding peptide as a targeting ligand for DNA-loaded NPs [49].

### 2.10. Insulin (INS) and Nnsulin-like Growth Factor (IGF) Receptors 

INS and IGF in physiological conditions exert a specific function—glucose metabolism, and cell growth and proliferation, respectively—whilst both receptors produce similar biological functions in cancer conditions, sharing a signaling pathway that plays an important role in cancer development and progression. For this reason, both receptors have emerged as targets for cancer therapy [50].

Far from being an exhaustive list of the different ligand-receptor strategies that have been exploited by Italian researchers and that will be described in this review, this chapter is intended to emphasize the fact that the search for good anticancer targets and targeting ligands continues and is strongly stimulated by the heterogeneous and adaptive nature of many tumor types.

## 3. Ten Years of Italian Research on Actively Targeted Nanocarriers

### 3.1. Polymer-Based Nanocarriers

**Figure 1:**
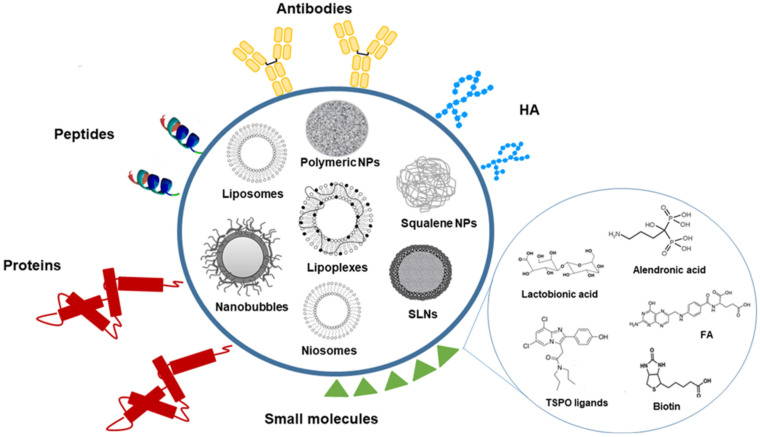
Schematic representation of most common actively targeted nanocarriers developed by Italian Technology research groups using ligand-based strategies over the last 10 years.

Polymer-based nanocarriers encompass a large variety of nano-sized colloids that are formed by natural or synthetic polymers into which a drug is either physically dispersed, dissolved or chemically bound to the polymer structure (Figure 1).

In most cases, the associated drugs are lipophilic, but hydrophilic compounds can also be incorporated by tuning the nanocarrier structure. Polymers have attracted much attention in the drug-delivery field thanks to the diversity of the available polymer sources, their composition and the synthesis procedures that allow the macromolecular structure to be engineered to be biocompatible, biodegradable and to contain derivatizable functional groups that are available for linkage to different homing agents. The accurate design of polymers influences the characteristics of the nanocarriers, such as their size, morphology, surface charge, drug loading mechanism and release mechanism [51].

Polymers are also frequently used to coat inorganic NPs to increase their biocompatibility and facilitate covalent coupling with specific targeting ligands. Nevertheless, as already mentioned, inorganic NPs are not considered in this review.

Concerning the use of FA as a targeting agent for natural polymer-based actively targeted nanocarriers, the vitamin was coupled to chitosan (CHIT) via an amide linkage. Methotrexate (MTX)-loaded NPs were then obtained using an ionotropic gelation technique. In the presence of high concentrations of reductive agents in cancer-cell microenvironments, these multifunctional NPs have shown redox-responsive drug release via the conjugation of L-cysteine to CHIT. The presence of FA moieties on targeted NPs provided higher selectivity towards FR-overexpressing HeLa cells, compared to untargeted nanocarriers [52].

Other stimuli-responsive nanosystems have been prepared from triblock copolymers formed of two terminal hydrophilic blocks (polyethylene glycol (PEG) and polyglycerolmethacrylate (poly-GMA)- and a central weakly basic block (polyimidazole-hexyl methacrylate (poly-ImHeMA)). In these polymerosomes, the central part was able to complex oligonucleotides, while the imidazole-containing side chains conferred pH-responsiveness to control carrier formation/disassembly. FA was linked to the copolymer, via an amide linkage, and a mixture of targeted and untargeted chains was used to prepare polymerosomes that were tested in vitro. The results showed a 5.5-fold higher uptake of double-strand-DNA-loaded folate-polymersomes in KB cells, compared to the untargeted ones [53,54].

A mixture of dextran (DEX) amphiphilic derivatives has been proposed as a means to obtain FA-targeted and pH/redox-responsive NPs. In more detail, DEX was conjugated to FA via esterification (DEXFA) and, separately, to PEG (DEXssPEGCOOH). The two derivatives self-assembled into NPs that could be destabilized in acidic pH and reducing media. In addition, encapsulated doxorubicin (DOX) showed a higher cytotoxic effect in FR+ cells [55].

When large molecules, such as PEG, are added onto the NP surface, the exposition of the targeting agent can be affected and, sometimes, hindered, especially for small ligands, such as FA. Thus, NPs have also been prepared via solvent displacement using mixtures of PEG-poly(ε-caprolactone) (PEG-PCL), with different PEG chain lengths (1.0 kDa or 2.0 kDa), and a FA-functionalized PEG-PCL (PEG 1.5 kDa), which was obtained via click-chemistry. The presence of shorter PEG chains allowed FA to be more exposed on the NP surface and assured, at the same time, reduced uptake by human macrophages and the higher uptake of targeted NPs by KB (FR+) rather than A549 (FR−) cells [56]. In another approach, the effective exposition of FA on NPs was evaluated by preparing core-shell FA-targeted NPs in a melting/sonication procedure using diblock copolymers of PCL and PEG and by adding (2-hydroxypropyl)-β-cyclodextrin (HPβ-CD) during the nanoassembly procedure. The interaction of HPβ-CD with the FA moieties allowed FA to be more highly exposed on the NP surface and, consequently, a higher uptake in cells that overexpress FR was achieved [57]. In a further development, FA exposure was obtained via the conjugation of the ligand to a PEG chain that was threaded with αCD in a single step reaction. In this original, selectively rotaxanated copolymer (Fol-PEG(αCD)-b-PCL), the presence of αCDs on the PEG chains led to a more extended conformation in the chains, which exposed the FA moieties after nanoprecipitation and, thus, promoted cellular uptake in FR-overexpressing cells compared to non-targeted NPs and the corresponding non-rotaxanated targeted carriers [58].

As far as the preparation of HA-decorated polymer NPs is concerned, different strategies can be exploited, such as HA-conjugate synthesis, the coating of NPs with HA, chemical cross-linking and ionotropic gelation.

Among natural polymers, CHIT has been thoroughly investigated and HA-decorated NPs have been produced via ionotropic gelation, by exploiting the electrostatic interactions between the positively charged amino groups of CHIT and negatively charged HA. The molecular weight and concentration of both the polymers, the CHIT degree of deacetylation, pH and temperature are the key parameters in formulation design, and they affect the NP physico-chemical characteristics and HA localization in the NP structure. A one-step inverse ionotropic gelation method was developed to optimize the deposition of HA on the NP surface [59]. The above-mentioned method was recently improved using microfluidics, and reproducible HA-decorated CHIT-based NPs with tailored-made narrow size distribution were obtained [60]. These NPs were avidly internalized in CD44+ human mesenchymal stem cells via CD44 receptor-mediated endocytosis, as demonstrated in competitive cellular binding experiments. Interestingly, the HA-CHIT NPs were able to load everolimus efficiently, and showed high cell-proliferation inhibition in CD44+ cells [60].

Albumin is another natural polymer that has been employed as a promising material in cancer therapy. Human serum albumin was used for the manufacturing of DOX-loaded micelle-like NPs [61]. They were prepared by the self-assembling of a HA-human serum albumin conjugate, obtained via their covalent coupling with 1-ethyl-3-(3-dimethylaminopropyl) carbodiimide (EDAC).

In addition, the insertion of cystamine moieties in the nanostructure using a cystamine-modified HA conferred redox-responsive functionalities. An increase in DOX release from albumin NPs was observed in the presence of a higher GSH concentration (10 mM), confirming the redox-responsive behavior. Moreover, the DOX-loaded NPs exhibited a CD44 receptor-mediated cell internalization mechanism in metastatic cancer (MDA-MB-231) cells, which was associated with a higher cytotoxic effect compared to the free drug.

Poly(lactic-co-glycolic acid) (PLGA) is one of the most commonly investigated polymers for anticancer DDS thanks to its biodegradability, biocompatibility and safety profile. In addition, it is FDA-approved for pharmaceutical applications and human administration. Concerning the development of actively targeted PLGA NPs through HA, this polysaccharide was often used as a coating material. For example, HA-coated PLGA NPs have been formulated for the delivery of sclareol, a phytochemical compound with anticancer properties [62]. PLGA NPs were obtained using the nanoprecipitation technique and then coated with high molecular weight HA (1500 kDa) via incubation. The HA coating increased the antitumor efficacy of the sclareol-loaded formulation against human breast cancer cells that expressed the HA receptors (MCF-7 and MDA-MB468), compared to the uncoated formulation, as it promoted cell interactions and the intracellular localization of the nanosystems.

Other studies have reported PLGA NPs being used as a core template and then surrounded by purposely designed shells that are endowed with the ability to target CD44 receptors. In particular, a polymer shell of low molecular weight HA (<10 kDa) and polyethyleneimine (PEI) has been built onto PLGA NPs using the layer-by-layer deposition technique, by exploiting the electrostatic interactions between oppositely charged polymers. HA/PEI-decorated NPs have demonstrated the capability to load docetaxel (DTX) to a great extent and release the encapsulated drug in a sustained manner [63]. Moreover, in vitro studies have demonstrated the enhanced uptake of rhodamine labeled HA/PEI-decorated PLGA NPs in CD44+ lung cancer cells and improved DTX cytotoxic activity, compared to the free drug. Intriguingly, this versatile nanostructure can be designed for the co-delivery of drug combinations by taking advantage of the shell and core properties.

A polymeric nanoplatform, based on a core of PLGA and a polymer shell of HA and PEI, has been proposed for the co-delivery of 5-fluorouracil (5-FU) and a plasmid that encodes the proapoptotic protein L3 (pL3) in the treatment of colon cancer [64]. The combined NPs enhanced 5-FU anticancer activity by increasing the chemosensitivity of human colon cancer cells. This tumor-selective chemosensitizer effect was due to the induction of apoptosis and the control of the drug efflux through the regulation of P-gp (P-glycoprotein) pump expression by L3 [58]. These HA-decorated double-coated PLGA NPs have recently been further investigated for the co-delivery of DTX and photosensitizers, such as disulfonate tetraphenyl chlorin and anionic porphyrin (TPPS4), to give targeted nanotools for combined chemo- and photodynamic therapy [65,66,67].

Moving now to the active targeting of polymer nano-sized carriers via biotin, mixed micelles made of Pluronics P123/F127 (synthetic triblock copolymers containing hydrophilic and hydrophobic regions) have been prepared using the thin-film hydration method to load repurposed drugs, such as the anthelmintic niclosamide and the photosensitizer verteporfin, that have been proposed for the treatment of multidrug resistant non-small lung cancer and locally advanced pancreatic cancer, respectively [68,69,70]. Biotin was first conjugated to Pluronic F127 via an ester bond. Then, the derivative was used to prepare the mixed micelles, which showed higher uptake in cancer cells than untargeted carriers and provided gradual drug release, at the same time.

Peptides and proteins have been conjugated to polymer NPs as well. In particular, an antiangiogenic peptide has been associated to an anticancer agent, namely DTX, in a combined approach, and tested in triple negative breast cancer cells. In more detail, DTX was incorporated into the inner part of PEG-PCL NPs using the nanoprecipitation technique, while an anti-vascular endothelial growth factor receptor 1 (anti-VEGFR1) hexapeptide was conjugated, via click-chemistry, to the PEG hydroxyl groups before NP formation. The combination of the loaded drug and the exposed peptide enhanced the antiangiogenic and in vivo anticancer effect in a xenografted mouse model of a MDA-MB231 tumor [71].

Antibody-engineered drug-loaded polymer NPs have been obtained from a different synthetic copolymer by conjugating rituximab, a chimeric monoclonal antibody that is targeted against the marker CD20, to PLGA. NPs were prepared using the oil-in-water single emulsion-solvent evaporation technique and loaded with Nutlin-3, a nongenotoxic activator of the p53 pathway. While Nutlin-3-loaded targeted and untargeted NPs did not show a significant difference in the activation of the p53 pathway in p53 wild-type JVM-2 leukemic cells in in vitro tests, actively targeted Nutlin-3-containing nanocarriers displayed the highest therapeutic activity in terms of survival rate in vivo [71]. Nutlin-3-PLGA NPs have also been formulated, via nanoprecipitation, and conjugated to the human monoclonal antibody against CD-138/Syndecan-1 (anti-Syndecan-1) to target primary effusion lymphoma. Again, targeted NPs showed increased selectivity compared to untargeted carriers [72].

In another approach, PLGA NPs that were loaded with curcumin (CUR) were prepared using the single emulsion solvent evaporation technique and then surface-coated with different molecules: CHIT for adhesion to the gastro-intestinal mucosa; wheat germ agglutinin for colon targeting; the GE11 peptide for colon tumor targeting. The NP surface was decorated with CHIT via coulombic interaction, since it is a polycationic mucoadhesive polysaccharide that adsorbs onto the negatively charged PLGA surface, and via covalent linkage for wheat germ agglutinin and GE11. Results showed that, depending on the coating, NPs were taken up by the cells via different mechanisms, notably by electrostatic interaction for CHIT and specific active mechanisms for both wheat germ agglutinin and GE11 [73]. GE11-PLGA NPs were also obtained via the nanoprecipitation of a blend (1:1 weight ratio) of two different PLGA-based polymers. In the first, GE11 was covalently coupled to PLGA, while, in the second, PLGA was functionalized with PEG. The obtained NPs showed low cytotoxicity, high stability in plasma and an efficient targeting ability towards EGFR-overexpressing cancer cells. Furthermore, GE11-PLGA NPs were able to incorporate a hydrophilic model drug into the particle matrix [74].

PLGA NPs have also been conjugated to synthetic ligands of the translocator protein 18 kDa (TSPO), a mitochondrial protein that is overexpressed in many cancer types. These ligands, characterized by a 2-phenyl-imidazo-[1,2-a]-pyridineacetamide structure, possess pro-apoptotic activity that was combined with the anticancer mechanism of 5-FU through the incorporation of the drug into NPs that were prepared via a quasi-emulsion solvent diffusion method. The results of the in vitro tests, carried out on C6 glioma cells that overexpressed TSPO, showed that a synergistic effect occurred [75]. The same ligands have been conjugated to fourth generation poly(amidoamine) (G(4)-PAMAM) fluorescent dendrimers and tested in the same cell line in the presence of various endocytosis inhibitors in order to investigate the cellular-uptake mechanism of the targeted nanocarriers. The dendrimers were found to be quickly internalized by pinocytosis and directed towards the mitochondria, as demonstrated by subcellular fractionation and confocal microscopy, together with competition studies [76]. The versatile G(4)-PAMAM dendrimer has also been linked to lactobionic acid, which targets the asialoglycoprotein receptor; after conjugation with a fluorescent moiety and loading with sorafenib, targeted dendrimers displayed higher binding and uptake ability in the asialoglycoprotein receptor-expressing human liver-cancer cell line, HepG-2, compared to non-expressing HLE cells [77].

The bisphosphonate, alendronate, has been conjugated onto the surface of PLGA NPs in order to target anticancer drugs (such as DOX) towards skeletal tumors [78]. In vitro assays on human osteoclasts showed that the active targeting of PLGA NPs with alendronate enables the antiosteoclastic effect of the conjugated bisphosphonate to contribute to the inhibition of tumor-associated bone degradation [79]. Conjugation with alendronate was also applied to dendrimers; heterobifunctional PEG was linked to paclitaxel (PTX) and alendronate, forming amphiphilic derivatives that self-assembled into nanocarriers that simultaneously possessed the binding affinity for the bone mineral hydroxyapatite and the cytotoxic activity of PTX against PC3 prostate-cancer cells [80].

#### Polymer-Shelled Nanobubbles (NBs)

NBs are another polymer nanoplatform that have been studied for the active targeted delivery of anticancer drugs. NBs are core-shell nanostructures consisting of a core, made up of a gas or a vaporizable compound, surrounded by a shell (Figure 1). They are considered to be the next generation of microbubbles, and they are already in clinical use as contrast agents for ultrasound (US). Unlike microbubbles, NBs have a prolonged circulation time and can extravasate from blood vessels, but remain visible to US imaging. They are therefore considered theranostic nanocarriers due to the double function of being US-triggered DDS and US-imaging agents. They have been proposed as promising vehicles for the delivery of drugs, genes and gases [81].

Polymer-shelled NBs have attracted great research attention due to the ability of the shell to enhance stability and be engineered with ligands for active targeting. Biodegradable polymers, either synthetic or natural, have been used as shell components and functionalized for ligand-receptor-mediated tumor targeting.

FA-conjugated PLGA-PEG NBs that encapsulate DOX have shown marked accumulation in tumor sites in a biodistribution study on breast-cancer-bearing balb-C mice [82]. Moreover, Lf that was conjugated to poly(l-lactic acid) (PLA) NBs has shown increased affinity in vivo in tumor-bearing mice [83].

Antibody-linked NBs have also been reported in the active targeting of anticancer drugs. PLGA NBs were bound to the humanized HuCC49DCH2 antibody, and were shown to be able to target the over-expressed TAG-72 antigen in vitro [84]. The conjugation of MTX-loaded NBs to monoclonal anti-HLA-G antibodies is another example that has given promising results both in vitro and in vivo [85].

NB technology has been developed and investigated in the Italian research scenario. Interestingly, purposely tailored CHIT-shelled NBs have been developed for the delivery of antagomiR17 as a therapeutic option for Burkitt lymphoma treatment, using nanoemulsion as the template. The targeted NBs were prepared via the conjugation of rituximab, by means of reductive amination, to the CHIT shell of the preformed NBs. The targeting agent promoted the internalization of antagomiR17-loaded NBs into CD20-expressing cancer B cells, and enabled the anticancer effect to be exerted. In fact, the inhibition of tumor growth was achieved after the administration of targeted NBs to a human-SCID model of Burkitt lymphoma, while no therapeutic effect was observed when using untargeted NBs [86].

In addition, CHIT-shelled NB formulations have been proposed as an innovative immunotherapeutic platform for the treatment of HER2+ breast cancer. NBs were conjugated with anti-CD11 antibodies to target dendritic cells (DCs), and loaded with a DNA vaccine. The targeted pHER2-loaded NBs were able to induce the activation of DCs and elicit a specific anticancer immune response, delaying tumor growth in vivo [87].

### 3.2. Lipid-Based Nanocarriers

Liposomes are spherical nanovesicles that consist of natural or synthetic phospholipid bilayers surrounding a water core (Figure 1). Liposomes are spontaneously formed when phospholipids are dispersed in water. As phospholipids are the major component of liposomes, they are nontoxic, biodegradable and biocompatible. Moreover, they are biologically inert, weakly immunogenic, and they have low intrinsic toxicity. They can encapsulate a wide variety of drugs. Whether the drug is encapsulated in the aqueous core or in the surrounding bilayer of the liposome is dependent on the characteristics of the drug and on the encapsulation process. In general, lipophilic drugs are entrapped almost completely in the lipid bilayer, hydrophilic compounds are encapsulated in their aqueous core and amphiphilic drugs partition between the lipid and aqueous phases, both in the bilayer and in the aqueous core [88,89]. A number of liposomal formulations are on the market for the treatment of different therapeutic applications [90].

Liposomes that co-encapsulate DOX and sorafenib and are decorated with the LinTT1 peptide, a specific molecule that targets the p32 protein, which is overexpressed by breast cancer and cancer associated cells, have been prepared and characterized [91]. The peptide was inserted onto the surface of preformed liposomes. The in vitro activity of the liposomes was evaluated in both positive estrogen receptor (MCF-7) and triple negative breast cancer (MDA-MB-231) cells in 2D and 3D cellular models. The results showed that the uptake and cytotoxicity of the LinTT1-functionalized liposomes was higher than that of plain liposomes and a combination of free drugs. Moreover, the LinTT1-functionalized liposomes were partly internalized and partly associated to the external cell membranes of primary human M2 macrophages, suggesting that there may exist a mechanism to modulate the penetration and accumulation of LinTT1-decorated liposomes in the central portion of the hypoxic area of tumors.

A new loading method, based on the use of a complexing agent already entrapped inside the liposome compartment (amino-lactose), has been developed for the efficient encapsulation of bortezomib (BTZ) in the aqueous core of liposomes with the aim of forming a boronic ester between the boronic acid moiety of BTZ and the hydroxyl groups of amino-lactose. A NGR-motif-containing peptide was then linked to the liposome surface as a targeting ligand for the tumor endothelial cell marker to target aminopeptidase N-positive tumor vessels [92]. The formulations were characterized in terms of encapsulation efficiency, drug release, size and Zeta-potential value. In vivo experiments were performed in orthotopic-neuroblastoma-bearing mice and showed that liposomal formulations provided a significant reduction in systemic adverse drug effects and that the presence of the peptide on the surface of liposomes improved the ability of BTZ to decrease neuroblastoma cell growth [93]. A similar approach was previously used for the preparation of liposomes that were decorated with NGR and encapsulated the apoptotic and antiangiogenic drug, fenretinide, which has been proposed as an adjuvant tool in the treatment of neuroblastoma [94].

Liposomes that can target bombesin (BN)-receptor-overexpressing tissues have been prepared for theranostic purposes. A new amphiphilic peptide derivative (MonY-BN) that contained the BN(7–14) peptide, the DTPA (diethylenetriaminepentaacetate) chelating agent, a hydrophobic moiety with two C18 alkyl chains and PEG spacers was synthesized using solid-phase methods. MonY-BN was added to the phospholipid during liposome preparation, and DOX was further encapsulated using the remote pH gradient method. Radiolabeled control liposomes were prepared by adding trace amounts of 111InCl3. Several analyses were performed to evaluate the liposome structure, drug-loading ability, in vitro cellular binding and cytotoxicity and in vivo therapeutic efficacy. The results showed that the simultaneous presence of both the peptide and the chelating agent should be able to provide the targeting ability and allow radiolabeling for diagnostic purposes. [95]. In order to enhance the activity of the system, the same group proposed a modified sequence of the BN(7–14) peptide that contained a modified BN sequence (BN-AA1) that should yield the desired high serum stability, high receptor affinity and act as a BN antagonist. The decorated liposomes showed targeting abilities and improved cytotoxicity in PC-3 cells and PC-3 xenograft-bearing mice. Although the results were quite similar to those obtained using the BN(7–14) peptide, the authors suggested that the AA1 antagonist sequence should provide safer working conditions for the further development of DDS [96]. The same research group proposed the use, as a targeting moiety, of a cyclic peptide, named Peptide R, which was identified as the most active of the recently proposed CXCR4 receptor antagonists. The chemokine receptor CXCR4 is expressed in immune cells and overexpressed in tumor cells, where, upon binding to its ligand CXCL12, it plays a critical role in invasion and metastasis in solid and hematological cancers and tumors [97]. DOX-loaded liposomes were then decorated with Peptide R, via conjugation of the previously thiolated peptide, and the maleimide-PEG chain on their surface. These liposomes were proposed for combination therapy; the in vitro test showed that they were able to deliver DOX efficiently and improve its cytotoxicity in CXCR4-expressing cell lines, while they were able to deliver the drug to melanoma lung metastases in vivo efficiently and reduce metastasis formation thanks to their antagonistic CXCR4 activity [98]. A similar conjugation method was used for the preparation of urotensin-II (UT)-decorated liposomes that encapsulated DOX and were able to bind the UT receptor overexpressed on colon, bladder and prostate cancer cells [99]. In this paper, the cysteine residue that was inserted into the peptide reacted with the PEG maleimide group on the liposome surface, and the targeting ability of the system was confirmed in in vitro tests on prostate- (DU145, PC3 and LNCaP) and colon-cancer cell lines (WIDR and LoVo). The cells showed different levels of UT-receptor expression, and the results demonstrated that the activity of the decorated liposomes was higher in the cells that overexpressed the receptor [100].

Acidity-sensitive vesicles (liposomes and niosomes) have been prepared by inserting, into the hydrophobic region of the nanovector, a conjugate of 1,2-distearoyl-sn-glycero-3-phosphoethanolamine (DSPE) lipids and pH (low) insertion peptides (pHLIP). Studies into the characteristics of the nanocarriers showed that the peptide is anchored and lies in the external surface. They should interact with target membranes at the acidic pH of the tumor microenvironment and release their content after bilayer rearrangement in low pH environments [101].

The same research group previously proposed an in vivo biodistribution study of fluorescently labeled pHLIP-coated niosomes in mice that bore 4T1 mammary tumors, showing significant tumor accumulation, compared to the non-targeted niosomes, and minimal kidney, liver and muscle accumulation [102].

The above-mentioned niosomes (non-ionic liposomes or non-ionic surfactant vesicles) are non-ionic surfactant-based vesicles that are able to self-assemble into unilamellar or multilamellar vesicles in aqueous media [103]. Non-ionic surfactants tend to orient themselves with the hydrophilic end facing the aqueous phase and the hydrophobic one facing inwards towards each other to form a closed bilayer structure, which encloses solutes in an aqueous solution (Figure 1). The formation of the closed bilayer structure is rarely spontaneous [104], and usually involves energy such as physical agitation or heat. Although not lipid-based, they are reported in this section due to their structural similarity with liposomes. Niosomes were first reported in the seventies in the cosmetics industry [105] but have been proposed as versatile and economic nanocarriers that are able to encapsulate a wide range of drugs, also thanks to their low cost, great stability and resulting ease of storing the non-ionic surfactants [106,107]. By varying the components, in terms of type of surfactant, composition and concentration, it is possible to change the features of the niosomes, including their size, surface charge and volume [108].

As already reported for polymer-based nanocarriers, HA has been proposed as a ligand to improve the targeting ability of liposomes and lipoplexes. For this purpose, conjugates between HA of different molecular weights and phospholipids have been prepared, characterized and employed for the preparation of actively targeted nanocarriers.

Low-molecular-weight HA (4.8 kDa and 12 kDa) has been reacted with the aminated lipid 1,2-dipalmitoyl-sn-glycero-3-phosphoethanolamine (DPPE) via reductive amination to produce conjugates that were added to the lipidic mixture during liposome preparation. Only one lipid molecule is linked to HA in these conjugates. In order to propose this system for the treatment of pancreatic adenocarcinoma (PDAC), which is characterized by the high expression of the HA-specific receptor CD44, liposomes were loaded with a lipophilic gemcitabine (GEM) prodrug. Liposomes were fully characterized in terms of size, surface charge and morphology. Further in vitro and in vivo studies showed that all of the liposome formulations were characterized by higher antitumoral activity than the free drug. The 12 kDa HA liposomes had the strongest efficiency, while non-conjugated liposomes and the 4.8 kDa HA liposomes were similarly active [109,110]. A novel system that is targeted towards pancreatic cancer stem cells, which are known to be responsible for resistance to standard therapy, has been developed to improve current therapies against PDAC. In particular, diethyldithiocarbamate-copper-containing, HA-decorated liposomes that were able to target the specific cancer stem cells marker CD44 receptor were prepared using the ion-gradient technique and fully characterized. Their antiproliferative effect was evaluated in pancreatic cancer stem cells that were either derived from PDAC cell lines or patients, and strongly increased anticancer activity was observed, paving the way for the development of nanomedicine-based cancer-stem-cell-targeting therapeutic approaches [111].

The HA-DPPE conjugate has also been used for the preparation of decorated liposomes that encapsulate a synthetic DOX that is conjugated with a H2S-releasing moiety that was demonstrated to be less cardiotoxic and more effective than DOX against Pgp-overexpressing osteosarcoma cells. HA liposomes showed a favorable drug-release profile and higher toxicity in vitro and in vivo than DOX or Caelyx (commercial DOX-loaded PEGylated liposomes) against Pgp-overexpressing osteosarcoma. Unlike DOX, HA-liposomes delivered the drug inside the endoplasmic reticulum (ER), inducing protein sulfhydration and ubiquitination, and activating an ER-stress pro-apoptotic response. HA-liposomes also sulfhydrated the nascent Pgp in the ER, reducing its activity [112].

Recently, the targeting ability of new conjugates that were obtained by reacting HA oligosaccharides (with a degree of polymerization, DP, of 4, 6 and 8) with a PEG-phospholipid moiety was evaluated. The conjugates were employed for the preparation of fluorescently labeled HA-DP-decorated liposomes that showed significantly higher (12- to 14-fold) cellular uptake in lung-cancer cell lines with high CD44 expression than in those with low CD44 expression, suggesting that the formulations underwent receptor-mediated entry. Moreover, uptake increased with increasing DP. HA-DP-decorated liposomes did not show cytotoxicity or inflammatory effects, suggesting that these conjugates may be used as biocompatible and effective tools for potential drug delivery to CD44+ cells [113]. The above-mentioned lipoplexes are lipid-based non-viral vectors that have been proposed as a means to transfer nucleic acids, such as plasmid DNA, siRNA and mRNA, to treat a wide range of disorders, from infectious and inherited diseases to cancer (Figure 1). They have some attractive features compared to viral vectors, and these include their easy production and chemical modification, low immunogenicity, safety and enhanced loading. They are composed of positively charged lipids that can interact and bind negatively charged nucleic acids. This electrostatic interaction between cationic non-viral vectors and the negative charged phosphate groups leads to a compaction in the system. Moreover, the presence of positive charges confers protection from nuclease attack and improves cellular uptake [114,115].

High-molecular-weight HA (1500 kDa) has been coupled to 1,2-dioleoyl-sn-glycero-3-phosphoethanolamine (DOPE) by means of an amidation reaction in which the amino group is randomly linked to the carboxylic residues of the polymer, and then introduced into the lipoplexes during preparation [116]. The lipoplexes were prepared via the addition of DNA to cationic liposomes, some of which contained the HA-DOPE conjugate, while others did not and were characterized. In vitro studies on breast cancer cells indicated that lipoplex cytotoxicity was not modified by the presence of the HA-DOPE conjugate, but its presence increased the transfection efficiency on CD44+ MDA-MB-231 cells compared to the CD44- MCF-7 line. The strong specificity of DNA targeting via the CD44 receptor using high-molecular-weight HA as the ligand was demonstrated by the same authors in the A549 lung-cancer cell line using a reporter plasmid that encoded the green fluorescent protein [117].

The HA-DOPE conjugate was also used for the preparation of lipoplexes for the delivery of anti-telomerase siRNA to CD44+ lung-cancer cells. The targeted lipoplexes displayed improved stability in the cell-culture medium and reduced cytotoxicity, without altering the binding or protection of siRNA [118]. In another study, the ability of HA-decorated siRNA lipoplexes was evaluated both in vitro and in vivo in a murine A549 metastatic lung-cancer model, using an anti-Luc siRNA. The potential of the system was demonstrated by the decrease in luciferase expression, which contrasts with the progressive increase observed when using non-modified lipoplexes [119]. The structure and morphology of these lipoplexes were investigated in detail using different techniques and approaches, including radioactive labeling, diameter and surface charge analyses, capillary electrophoresis, cryo-TEM microscopy, SAXS and surface plasmon resonance [120].

Tf has been used to decorate liposomes, niosomes and self-assembling lipid NPs. The anticancer activity of the antimalarial sesquiterpene lactone artemisinin was explored by encapsulating the compound in liposomes. Drug loading in nanocarriers was also explored to improve its stability and solubility. Liposomes were then decorated with Tf to obtain specific targeting towards cancer cells that overexpress TfRs. Tf was inserted onto the surface of preformed liposomes via a coupling reaction between DSPE-PEG-COOH, used as a linker lipid, and the free amino groups of the protein in the presence of EDAC. The plain and decorated liposomes were characterized in terms of size, drug-entrapment efficiency, Tf-coupled coupling moieties and stability. The targeting ability of Tf liposomes was assessed in in vitro studies (cell uptake and cytotoxicity) on the human colon cancer cell line HCT-8, which is characterized by the overexpression of TfR [121].

Novel hybrid self-assembling NPs (PLCaPZ NPs), based on calcium phosphate and lipids, were developed to deliver zoledronic acid (ZOL) to different tumor models and displayed superior delivery efficiency than PEGylated liposomes [122]. In order to improve the targeting ability of PLCaPZ NPs towards glioblastoma, Tf was added during PLCaPZ NP preparation to obtain Tf-PLCaPZ NPs. The structural characterization of the system was performed using different techniques, including small angle neutron scattering, X-ray scattering and cryo-electron transmission microscopy [123]. The efficacy of Tf-PLCaPZ NPs was evaluated in different glioblastoma cell lines and in an animal model of glioblastoma and was compared with PLCaPZ NPs and free ZOL. In vitro results in LN229 cells showed significant uptake and the improved cytotoxicity of Tf-PLCaPZ NPs. The results obtained in mice xenografted with U373MG revealed that Tf-PLCaPZ NPs had the highest anticancer activity [124,125]. Thiolated Tf, obtained via derivatization with 2-iminothiolane (Traut’s reagent), was coupled with the preformed stable nucleic acid lipid vesicles (SNALPs) that contained DSPE-PEG-maleimide, forming a stable thioether bond between lipid vesicles and the ligand. miR-34a was encapsulated to target TfR-overexpressing multiple myeloma cells. The vesicles demonstrated the ability to inhibit tumor growth in vivo, and Tf-conjugation resulted in a significant prolongation of mouse survival [126].

Niosomes have been employed as nanocarriers for the active targeting of DOX in breast cancer cells that overexpress TfRs (MCF-7 and MDA-MB-231). Niosomes were prepared using, among other components, an opportunely modified Pluronic L64 surfactant that can react with the protein. In particular, free Tf amino groups were linked to the carboxylic groups of the carrier on the external surface of the niosomes in the presence of EDAC. Niosomes were found to be regular in shape and homogeneous in size, and DOX was easily encapsulated into them, without affecting vesicle morphology. The in vitro test on fluorescently labeled Tf niosomes indicated that the nanocarriers were mainly taken up by TfR-mediated endocytosis. When DOX was loaded, Tf niosomes showed a significant reduction in viability in a dose- and time-related manner [127]. The same group proposed an evolution of this approach by coupling either Tf, FA or a combination of the two for the delivery of either DOX and CUR, or DOX and quercetin, into multifunctional niosomes. In vitro experiments showed that the niosomes loaded with DOX/CUR had the highest cytotoxic activity, confirming the synergistic effects of the combination. Finally, the reduction in cell viability followed the trend; Tf, Tf-FA- and FA-niosomes. These results may be due to the condensation reaction between the carboxylic groups of the surfactant and the two different amino groups of FA, one of which may reduce the ligand-receptor recognition required for cellular uptake [128].

Recent work by Italian research groups that used FA as a targeting ligand has not only reported the preparation of liposomes, but also other structures to improve the targeting ability of anticancer drugs.

In particular, supramolecular vesicular aggregates (SVAs) have been prepared via the self-assembly of liposomes and FA-conjugated polyasparthydrazide co-polymers that encapsulated GEM. The SVAs were then characterized [129]. The authors previously demonstrated that SVAs do not alter the structure of the vesicles, allowing the systems to deliver the entrapped drug efficiently. In vivo experiments in mice bearing MCF-7 human xenografts, used as a breast-cancer model, showed that FA-decorated SVAs were more active than GEM, whether they were free or loaded into liposomes.

Another research group has compared the cytotoxicity and selectivity of DOX-containing liposomes and DOX that was conjugated to pullulan-based polymeric nanocarriers, both with or without FA [130]. The FA-decorated liposomes were prepared via the post insertion of the FA-DSPE (1,2-distearoyl-sn-glycero-3-phosphorylethanolamine) conjugate on the Caelyx surface. The targeting ability of FA-containing DDS was demonstrated in vitro. The in vivo experiments in FR-overexpressing human cervical carcinoma KB tumor-bearing mice showed that both systems were active and able to reduce DOX-induced cardiotoxicity. This work compared two receptor-targeted ligand-bearing systems, polymer therapeutics versus nanoparticulate systems, and evaluated them in the same mouse tumor model at several dosing regimens.

FA-decorated liposomes that contained a synthetic DOX that was conjugated with a nitric oxide (NO)-releasing group, which have demonstrated the ability to overcome resistance by inducing the NO-mediated inhibition of P-glycoprotein (Pgp), were prepared and characterized. FA-liposomes were prepared using two different methods: FA-DSPE conjugate insertion on liposomes via hydration (FA on both faces of the bilayer) and using the post-insertion method (FA only on the external face of the bilayer). The formulations were compared in terms of their technological features and in vitro behavior. The results showed that FA-liposomes are internalized in a FR-dependent manner and achieve maximal antitumor efficacy against FR+/Pgp positive cells. In vivo experiments showed that liposomes reduced the growth of FR+/Pgp-positive tumors and prevented tumor formation in mice, whereas DOX and Caelyx failed. FA-liposome cardiotoxicity was comparable to that of Caelyx [131].

Etoposide has been encapsulated into liposomes that were functionalized with anti-GD2 mAb 3F8, which has demonstrated the ability to display anti-tumor effects via antibody-directed cellular cytotoxicity and complement-mediated cytotoxicity [132]. GD2 is a disialated ganglioside that is expressed on tumors of neuroendocrine origin (melanoma, osteosarcoma and neuroblastoma) [133]. Thiolated Ab was conjugated via a reaction with DSPE-PEG-maleimide that was post inserted into the external surface of the liposomes. The liposomes were fully characterized, and in vitro studies showed that immunoliposomes entered the targeted cells via clathrin-dependent uptake, and a correlation between cellular GD2 levels and the ability to target cells using etoposide-containing immunoliposomes was observed [134].

Immunoliposomes that encapsulated 5-FU and were decorated with an Ab against the Frizzled 10 (FZD10) protein, which is a cell surface receptor belonging to the FZD-protein family and is overexpressed in colorectal cancer cells, were prepared and characterized. The primary amine groups of the Ab were conjugated to liposomes that bore carboxylic groups via crosslinking chemistry. In vitro studies showed that the cytotoxic activity of immunoliposomes was enhanced compared to the free drug and untargeted liposomes, indicating that the use of FZD10 protein as a novel, effective target for colorectal cancer should be explored [135].

PEGylated lipoplexes that were either plain or conjugated to a mAb directed towards primary effusion lymphoma cells and that contained a model oligonucleotide have been prepared and characterized. The in vitro transfection efficiency was evaluated on BCBL-1 cells, and the mAb-containing lipoplexes displayed a significant increase in the transfection rate and the localized internalization of the oligonucleotide, with respect to the plain analogues [136]. The same group developed combined approaches, transmission electron and atomic force microscopic techniques, to evaluate and identify the modification on the surface of liposomes deeply [137].

Niosomes that were functionalized with the glucose-derivative N-palmitoylglucosamine were developed as a potential brain-targeted delivery system [138]. Vesicles were loaded with DOX and fully characterized in terms of size, surface charge and morphology. Preliminary in vivo studies, performed on rats, showed that the glucose-targeted vesicles significantly reduced the heart accumulation of the drug, prolonged its blood circulation and were able to improve its brain delivery significantly, with respect to free DOX. The exact mechanism of transport is not completely known, but the author indicated some hypotheses.

A novel synthetic non-peptide oligo-arginine cell-penetration enhancer (CPE), formed of four arginine units conjugated to a 2,2-bis(hydroxymethyl)-propionic acid (bis-MPA)/2,2-bis(aminomethyl)propionic acid (bis-AMPA) polyester dendron that terminated in 1,2- distearoyl-3-azidopropane (DAG-Arg4), has been proposed for liposome bilayer insertion. Thanks to its dendritic structure, DAG-Arg4 has shown higher charge density and increased cellular uptake than linear polypeptides and has a hydrophobic residue that anchors it to the liposome bilayer [139]. Liposomes that encapsulated DOX and were decorated with DAG-Arg4 have been tested in mice with an orthotropic model of breast cancer (4T1-Luc murine mammary carcinoma), using several analytical approaches. DAG-Arg4-decorated liposomes showed reduced tumor and spleen accumulation, similar liver accumulation and higher lung accumulation than plain liposomes. However, their in vivo anticancer activity was the highest and was probably due to the intra-tumoral permeability that is mediated by the CPE on the liposome surface [140], which has been proposed as a targeting ligand for hepatocellular carcinoma delivery [141]. The thyroid hormone triiodothyronine (T3), which has a demonstrated role in hepatocellular carcinoma suppression, was encapsulated in plain and Lf-decorated liposomes. Lf was thiolated and further conjugated onto the surface of the preformed liposomes, which contained the DSPE-PEG-maleimide lipid. In vitro studies on target cells (FaO, HepG2 and SKHep) showed that Lf-liposomes had higher cell binding and cellular uptake, with high affinity to multiple receptors on the cell surface, than the non-decorated analogues. Cell-viability tests, performed using hepatoma cell lines, demonstrated that all of the formulations had very low toxicity, but indicated that liposomes may ensure specific and sustained drug delivery and a reduced therapeutic dose, thus avoiding the side effects that are associated to T3 treatment [142].

Nanoliposomes that were decorated with a thyroid-stimulating hormone (TSH) were prepared in order to target specifically the TSH receptor (TSHr), which is a glycoprotein expressed in the plasma membranes of thyrocytes, and is maintained in most thyroid pathologies and also present in the majority of less differentiated and more aggressive tumors [143]. Decorated vesicles were prepared by conjugating the cysteine residue in TSH with a thiol-activated moiety that is present on the surface of the nanoliposomes, forming a disulfide bond. The nanoliposomes were fully characterized and showed an increased uptake in cells with TSHr, in comparison with plain ones, as well as higher levels of accumulation in normal thyroid tissue and in subcutaneous thyroid tumors. Finally, GEM-loaded TSH-nanoliposomes displayed a significant improvement in in vitro and in vivo anticancer activity [144].

Solid Lipid Nanoparticles (SLNs) are solid nanosystems based on lipid matrices that are solid at body temperature, such as triglycerides, fatty acids, phospholipids and waxes [145]. Thanks to several formulation strategies, it is possible to incorporate both lipophilic and hydrophilic drugs and to scale-up production methods [146,147]. As SLNs have demonstrated biocompatibility and the capability to improve drug bioavailability by increasing their diffusion through biological membranes in several studies [148], the development of actively tumor-targeted SLN formulations is currently in progress [149].

In the Italian landscape, SLNs have been proposed as nanosystems that are actively targeted towards brain tumors. Endogenous and chimeric ligands have been directly or indirectly bound to drug-loaded nanocarriers to obtain systems that can recognize brain capillary endothelial cells and cerebral tumoral cells, making them a promising strategy in oncology. Some researchers have studied SLNs as possible active targeting carriers for the treatment of glioblastoma multiforme, and have selected MTX as a cytotoxic drug against rat F98 glioma cells and prepared the lipophilic derivative di-dodecyl-MTX (ddMTX) to be loaded into SLNs [150]. ddMTX-loaded behenic acid-SLNs were prepared using the coacervation method, and active targeting was obtained using apolipoprotein E (ApoE), a chimera peptide that can bind the very low-density lipoprotein (VLDL) receptor and anchor to lipid surfaces via simple electrostatic interactions. ddMTX-loaded SLNs were shown to have antiproliferative activity that is mediated by the induction of apoptotic death in F98 glioma cells. Biodistribution studies in healthy rats demonstrated the superiority of the ApoE-conjugated formulation. No difference was observed in brain uptake between healthy rats and glioma models, and this is probably because the particle size exceeds the fenestrations of glioma-altered BBB. On the other hand, differences in tumor growth rate (measured through MRI) and apoptosis were observed in the control and treated rats.

With the aim of improving the data obtained in the work described above, stearic acid and behenic acid-SLNs were loaded with ddMTX and surface-functionalized with two different proteins, Tfr and INS, whose receptors are overexpressed on the BBB [151]. The surface-functionalization was obtained via the addition of a lipophilic (ST-MBS) or PEGylated (ST-PEG-MBS) N-octadecil-3-maleimido-benzamide linker, which was added to the fatty-acid micellar solution prior to acidification, while the proteins were thiolated and grafted onto the exposed maleimide moiety on SLN surfaces. Both linkers made it possible to graft proteins onto SLN surfaces, although ST-MBS allowed a higher protein amount to be bound. In vitro permeation through hCMEC/D3 cells showed significantly increased permeation through the hCMEC/D3 cells’ monolayer, compared to free MTX, for both non-functionalized and functionalized SLNs. Only SLNs that were functionalized with ST-PEG-MBS showed a significant increase in accumulation in the brain in an in vivo study, although a higher protein amount was grafted with ST-MBS. Furthermore, capillary depletion showed no endothelial-cell internalization for ST-MBS-grafted SLNs, and good uptake for ST-PEG-MBS-grafted SLNs. The authors highlighted the importance of a PEG spacer between the SLN surface and the targeting protein for interactions with the receptor.

Squalene Nanoparticles. The “squalenoylation” approach allows drugs to be conjugated to natural and biocompatible SQ to produce tailored derivatives that are able to self-assemble in water. The formed SQ nanoassemblies are multifunctional NPs with high drug loading that, in most cases, display an improved pharmaceutical profile compared to the parent compound [152]. In a paper on this topic, the anticancer agent GEM was conjugated to 1,1′,2- trisnorsqualenic acid via the acylation of the C-4 nitrogen atom of the cytosine nucleus. The hexapeptide CKAAKN was conjugated to SQ and then associated to GEM-SQ by co-nanoprecipitation to target GEM-SQ actively towards pancreatic cancer cells. The obtained targeted NPs have demonstrated that they are not only able to interact specifically with both cancer cells and angiogenic vessels, but also to favor the normalization of the vasculature. Moreover, in vivo tests in RIP-Tag2 mice showed that there was a higher reduction in tumor volume for CKAAKN-targeted SQ NPs than for both the free drug and untargeted nanocarriers [153]. Since the strategy for coupling the ligand to the NPs is of paramount importance, a study was performed to evaluate the impact of the conjugation strategy on the in vitro targeting ability of peptide-decorated GEM-SQ NPs. In more detail: In one approach, the CKAAKN peptide was first reacted with a SQ derivative and then co-nanoprecipitated with GEM-SQ; in a second approach, the peptide was conjugated to the surface of preformed GEM-SQ NPs. A higher binding ability and better specific avidity towards the peptide receptor were displayed by NPs that were prepared via the co-nanoprecipitation of the two SQ derivatives [154].

Most ligand classes and the strategies used to target the described polymer- and lipid-based nanocarriers actively are summarized in Table 1, together with their specific receptors and cancer types.

**Figure 2:**
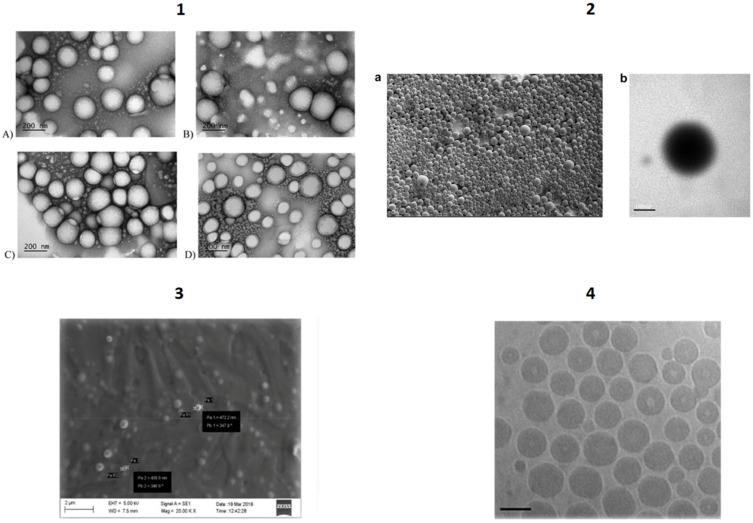
TEM/SEM images of polymeric NPs, NBs, SLNs, SQ NPs. **1.** TEM images of uncoated and coated CUR-PLGA NPs. Scale bar = 200 nm. (A) Uncoated CUR-PLGA NPs, (B) 1% CHIT-CUR-PLGA NPs, (C) wheat germ agglutinin-CUR-PLGA NPs and (D) GE11-CUR-PLGA NPs [73]. Permitted reproduction under the terms and conditions of the Creative Commons Attribution (CC BY) license (https://creativecommons.org/licenses/by/4.0/, accessed on 15 May 2021). **2.** (a) SEM image of mAbHLA-G/MTX/PLGA NBs; (b) TEM image of mAbHLA-G/MTX/PLGA NBs. Scale bar = 100 nm. Image adapted with permission [85]. Copyright Elsevier 2014. **3.** SEM image of bevacizumab-loaded SLNs. Scale bar = 2 µm. Image reproduced with permission from [155]. Permitted reproduction under the terms and conditions of the Creative Commons Attribution (CC BY) license (https://creativecommons.org/licenses/by/4.0/, accessed on 15 May 2021) **4.** Cryogenic TEM image of GEM-SQ/CKAAKN-SQ NPs. Scale bar = 100 nm. Reproduced with permission from [153]. Copyright Elsevier 2014.

**Figure 3:**
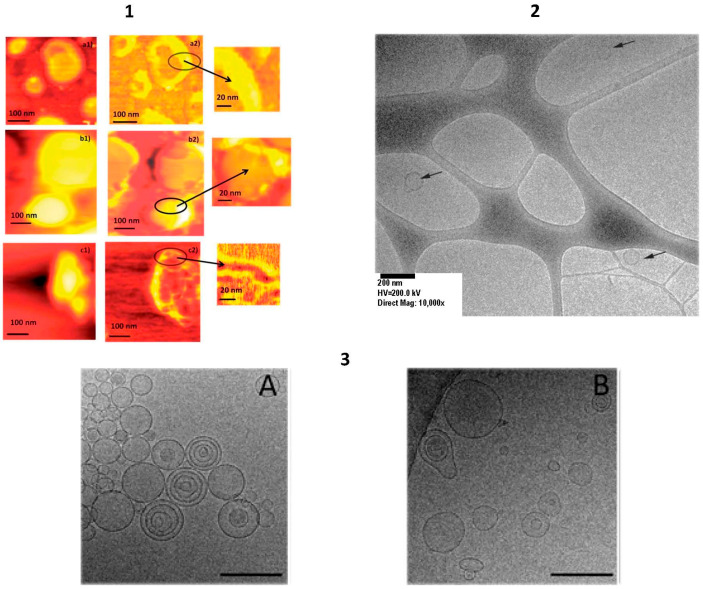
AFM/TEM images of liposomes, niosomes and lipoplexes. **1.** AFM images of different liposomes. ‘‘Height images’’ (1) and ‘‘phase images’’ (2) of cationic liposomes (a), classic pegylated liposomes (b) and immunoliposomes (c). Magnification of phase images; arrows indicate the detailed on liposomal surface. Image adapted with permission [137]. Copyright Taylor & Francis 2015. **2.** Representative cryo-TEM images of Span20 niosomes coated with pH (low) insertion peptide. Scale bar = 200 nm [101]. Permitted reproduction under the terms and conditions of the Creative Commons Attribution (CC BY) license (https://creativecommons.org/licenses/by/4.0/, accessed on 15 May 2021) **3.** Cryo-TEM images of (A) HA-liposomes, (B) HA-lipoplexes. Scale bar = 200 nm. Reproduced with permission from [120]. Copyright American Chemical Society, 2015.

In Figure 2 and Figure 3, SEM/TEM/AFM images of different NP typologies described in the present paper are reported.

## 4. Conclusions

Many Italian researchers, including chemists, engineers and physicists, have been working for years on the development of new nanotechnology-based DDS, and especially on the synthesis of innovative materials and the development of new linking strategies that can overcome the limitations of conventional anticancer therapies. Without wishing to devalue these crucial contributions, this review focuses on research activity carried out, since 2010, in Italian academic Pharmaceutical Technology laboratories on actively targeted NPs for cancer management. Indeed, the recent Italian research panorama on actively targeted NPs for cancer therapy has expanded significantly, generating promising results in vitro and in vivo, and this is also thanks to the continuous drive to develop fruitful multidisciplinary collaborations with national and international research groups.

Taking together the reported studies, we have observed constant evolution in the development of ligands, with a progressive shift from small molecules to macromolecules. Furthermore, ever-growing attention is being paid to optimizing the number of targeting agent molecules and/or ligand exposure on the nanocarrier surface using various decoration strategies.

In terms of nanocarrier types, liposomes are a consolidated approach as various surface functionalization strategies have been adopted with different targeting ligands, including FA, peptides and Ab. Many anticancer agents have been loaded into the developed liposomes and successfully tested for their efficacy both in vitro and in vivo. As some PEGylated liposomal formulations of DOX are already commercially available, it cannot be excluded that the intelligent design of some actively targeted liposomes, in terms of improving their selectivity towards tumor cells and the tumor microenvironment, might hasten their clinical translation.

Actively targeted polymer-based nanocarriers are a highly heterogeneous group of structures that have been developed starting from the accurate design of polymer synthesis and functionalization in order to tune matrix characteristics precisely. Moreover, a great variety of traditional and novel preparation methods has been proposed. The strategies adopted to furnish these nanocarriers with tumor targeting mainly exploit FA decoration, by optimizing the vitamin’s surface exposition, and the use of HA-decorated polymers.

Finally, the development of actively targeted SQ-NPs, SLNs and NBs in cancer therapy is an interesting “niche” research field for some Italian groups, and one that seems to be destined to improve the high physiological compatibility of these nanocarriers constantly and the preliminary in vitro and in vivo results.

In conclusion, it is to be expected that ongoing research developments and future perspectives will further optimize the safety, effectiveness and selectivity of the described actively targeted nanocarriers for the future translation of this approach to the clinic. Nevertheless, it must be underlined that, to achieve efficient clinical translation and to allow an actively targeted nanocarrier to reach patient beds, additional multidisciplinary research and established collaborations between academia, industry and regulatory bodies will be required.

## Figures and Tables

**Table 1:** Ligand-based actively tumor-targeted nanocarriers for the receptor-directed drug-targeting of various types of cancer over the last 10 years of Italian research.

Ligand	Nanosystem	Conjugation Strategy	Drug/Payload	Receptor	Cancer Type	Ref.
**Small molecules**						
FA	Polymeric NPs (CHIT)	amide bond	MTX	FR	cervical adenocarcinoma	[52]
FA	Polymerosomes	amide bond	ds-DNA and ds-siRNA	FR	cervical carcinoma, breast adenocarcinoma	[53,54]
FA	Polymeric NPs (DEX)	ester bond	DOX	FR	breast adenocarcinoma breast	[55]
FA	Polymeric NPs (PCL)	amide bond	fluorescent probe (Nile red)	FR	cervical carcinoma, lung carcinoma	[56,57,58]
biotin	Micelles	ester bond	niclosamide	biotin receptor	lung carcinoma	[68]
biotin	Micelles	ester bond	verteporfin	biotin receptor	prostate cancer, breast adenocarcinoma	[69]
TSPO ligands	Polymeric NPs (PLGA)	ester bond	5-FU	TSPO	glioma	[75]
TSPO ligands	G(4)-PAMAM dendrimers	amide bond	fluorescent probe (fluorescein)	TSPO	glioma	[76]
lactobionic acid	G(4)-PAMAM dendrimers	amide bond	sorafenib	asialoglycoprotein receptor	liver cancer	[77]
alendronate	Polymeric NPs (PLGA)	amide bond	DOX	bone hydroxyapatite	bone cancer	[78]
alendronate	PEG dendrimers	amide bond	PTX	bone hydroxyapatite	prostate cancer	[80]
FA	SVAs	covalent bond (FA conjugated polyasparthydrazide)	GEM	FR	breast cancer	[129]
FA	Liposomes	covalent bond (FA-DSPE conjugate post insertion)	DOX	FR	cervical carcinoma	[130]
FA	Liposomes	covalent bond (FA-DSPE conjugate in mixture and post insertion)	a synthetic DOX conjugated with nitric oxide (NO)-releasing group	FR	breast cancer	[131]
**Peptides and proteins**						
GNQWFI peptide	Polymeric NPs (PCL)	amide bond	DTX	VEGFR1	breast adenocarcinoma	[70]
GE11 peptide	Polymeric NPs (PLGA)	amide bond	CUR	EGFR	colon adenocarcinoma	[73]
GE11 peptide	Polymeric NPs (PLGA)	amide bond	model drug (myoglobin)	EGFR	lung carcinoma	[74]
LinTT1 peptide	Liposomes	covalent bond on the surface of preformed liposomes	DOX and sorafenib	p32 protein	breast cancer	[91]
NGR motif-containing peptide	Liposomes	covalent bond on the surface of preformed liposomes	BTZ	aminopeptidase N-positive tumor vessels	neuroblastoma	[93]
NGR motif-containing peptide	Liposomes	covalent bond on the surface of preformed liposomes	Fenretinide	aminopeptidase N-positive tumor vessels	neuroblastoma	[94]
BN(7–14) peptide	Liposomes	amphiphilic peptide derivative mixed with the other lipids	DOX and 111InCl3	BN receptors	prostate adenocarcinoma	[95]
BN-AA1	Liposomes	amphiphilic peptide derivative mixed with the other lipids	DOX	BN antagonist	prostate cancer	[96]
Peptide R	Liposomes	covalent bond on the surface of preformed liposomes	DOX	CXCR4 receptor-antagonist	melanoma lung metastasis	[98]
UT-II	Liposomes	covalent bond on the surface of preformed liposomes	DOX	UT-receptor	colon, bladder and prostate cancer	[100]
Tf	Liposomes	inserted on the surface of preformed liposome	artemisinin	TfR	colon cancer	[121]
Tf	hybrid self-assembling NPs	pre-formed cationic liposomes mixed with Tf	ZOL	TfR	glioblastoma	[124,125]
Tf	SNALPs	covalent bond on the preformed NC	miR-34a	TfR	mieloma	[126]
Tf	Niosomes	covalent bond on the external surface of the niosomes	DOX	TfR	breast cancer	[127]
Tf and FA	Niosomes	covalent bond on the external surface of the niosomes	DOX and CURC or DOX and quercetin	TfR	breast cancer	[128]
CPE (Arg4-DAG)	Liposomes	association to the liposome bilayer	model drugs (calcein or BSA)		cervical cancer	[139]
CPE (Arg4-DAG)	Liposomes	association to the liposome bilayer	DOX		breast cancer	[140]
Lf	Liposomes	covalent bond on the surface of preformed liposomes	T3	multiple receptors	hepatocellular carcinoma	[142]
TSH	Nanoliposomes	covalent bond	GEM	TSHR	thyroid carcinoma	[144]
ApoE	SLNs	electrostatic interaction	MTX derivative	VLDL	glioblastoma multiforme	[150]
Tf/INS	SLNs	covalent bond	MTX derivative	TfR/HIR	glioblastoma multiforme	[151]
CKAAKN peptide	SQ NPs	thioether bond	GEM	frizzled receptors	pancreatic cancer	[153]
CKAAKN peptide	SQ NPs	thioether bond	GEM	frizzled receptors	pancreatic cancer	[154]
**Antibodies**						
rituximab	Polymeric NPs (PLGA)	amide bond	Nutlin-3	CD20	chronic lymphocytic leukemia	[71]
rituximab	Polymeric NBs (CHIT)	reductive amination	antagomir17	CD20	Burkitt limphoma	[86]
Anti-CD11c or anti-CD1a monoclonal antibody	Polymeric NBs (CHIT)	reductive amination	plasmid coding for HER2	murine DC marker CD11c or human DC marker CD1a	breast cancer	[87]
anti-Syndecan-1 antibody	Polymeric NPs (PLGA)	amide bond	Nutlin-3	CD-138/Syndecan-1	lymphoma	[72]
mAb 3F8	Liposomes	covalent bond on the surface of preformed liposomes	etoposide	anti-GD2	neuroblastoma, cervical carcinoma, breast carcinoma, melanoma, osteosarcoma	[134]
Ab against Frizzled 10 (FZD10) protein	Liposomes	covalent bond on the surface of preformed liposomes	5-FU	anti-Frizzled 10 protein (FZD10)	colorectal cancer	[135]
**Hyaluronic acid**						
HA (HMW)+50:56	Polymeric NPs (CHIT)	ionotropic gelation	everolimus	CD44	human mesenchymal stem cells (hMSCs)	[59,60]
HA (LMW)	Micelle-like albumin NPs	amide bond	DOX	CD44	breast adenocarcinoma	[61]
HA (HMW)	Polymeric NPs (PLGA)	physical adsorption	sclareol	CD44	breast cancer	[62]
HA (LMW)	Polymeric NPs (PLGA)	layer-by-layer deposition	DTX	CD44	lung cancer	[63]
HA (LMW)	Polymeric NPs (PLGA)	layer-by-layer deposition	5-FU/plasmid pL3	CD44	colon cancer	[64]
HA (LMW)	Polymeric NPs (PLGA)	layer-by-layer deposition	DTX/photosensitizer (disulfonate tetraphenyl chlorin)	CD44	breast cancer and cervix cancer	[65,66]
HA (LMW)	Polymeric NPs (PLGA)	layer-by-layer deposition	DTX/photosensitizer (tetrasodium-meso-tetra- (4-sulfonatophenyl)porphyrin)	CD44	breast cancer	[67]
HA (LMW)	Liposomes	HA-phospholpid conjugate inserted in liposome	GEM lipophilic prodrug	CD44	pancreatic cancer	[109,110]
HA (LMW)	Liposomes	HA-phospholpid conjugate inserted in liposome	diethyldithiocarbamate-copper	CD44	pancreatic cancer	[111]
HA (LMW)	Liposomes	HA-phospholpid conjugate inserted in liposome	synthetic DOX conjugated with a H2S-releasing moiety	CD44	osteosarcoma	[112]
HA oligosaccharides	Liposomes	conjugate inserted in liposome	fluorescently labeled	CD44	lung cancer	[113]
HA (HMW)	Lipoplexes	HA-phospholpid conjugate inserted during lipoplexes formation	plasmid DNA	CD44	breast cancer	[116]
HA (HMW)	Lipoplexes	HA-phospholipid conjugate inserted during lipoplexes formation	plasmid DNA	CD44	lung cancer	[117]
HA (HMW)	Lipoplexes	HA-phospholipid conjugate inserted during lipoplexes formation	anti-telomerase siRNA	CD44	lung cancer	[118]
HA (HMW)	Lipoplexes	HA-phospholipid conjugate inserted during lipoplexes formation	anti-Luc siRNA	CD44	metastatic lung cancer	[119]

## Abbreviations

ApoE	apolipoprotein E
BBB	blood brain barrier
BN	bombesin
BTZ	bortezomib
CPE	cell-penetration enhancer
CUR	curcumin
CD	cyclodextrin
DC	dendritic cell
DDS	drug delivery system
DEX	dextran
DOPE	1,2-dioleoyl-sn-glycero-3-phosphoethanolamine
DOX	doxorubicin
DPPE	1,2-dipalmitoyl-sn-glycero-3-phosphoethanolamine
DSPE	1,2-distearoyl-sn-glycero-3-phosphoethanolamine
DTX	docetaxel
EDAC	1-ethyl-3-(3-dimethylaminopropyl)carbodiimide
EGFR	epidermal growth factor receptor
EPR	enhanced permeability and retention
FA	folic acid
FR	folate receptor
5-FU	5-fluorouracil
FZD10	frizzled 10 protein
GEM	gemcitabine
HA	hyaluronic acid
HEGFR	human epidermal growth factor receptor
HPβ-CD	2-(hydroxypropyl)-β-cyclodextrin
INS	insulin
Lf	lactoferrin
mAb(s)	monoclonal antibody(ies)
MDR	multidrug resistance
MTX	methotrexate
NB(s)	nanobubble(s)
NP(s)	nanoparticle(s)
PCL	poly(ε-caprolactone)
PDAC	pancreatic adenocarcinoma
PEG	polyethylene glycol
PEI	polyethyleneimine
PLGA	poly lactic-co-glycolic acid
PLA	poly lactic acid
PSMA	prostate-specific membrane antigen
PTX	paclitaxel
SLN(s)	solid lipid nanoparticle(s)
SQ	squalene
SVA(s)	supramolecular vesicular aggregate(s)
T3	thyroid hormone triiodothyronine
Tf	transferrin
TfR	Tf receptor
TSH	thyroid-stimulating hormone
US	ultrasounds
UT	urotensin-II
VLDL	very low-density lipoprotein

## References

[1] Kumari P., Ghosh B., Biswas S. (2015). Nanocarriers for cancer-targeted drug delivery. J. Drug Target..

[2] Senapati S., Mahanta A.K., Kumar S., Maiti P. (2018). Controlled drug delivery vehicles for cancer treatment and their performance. Signal. Transduct. Target. Ther..

[3] Gu W., Meng F., Haag R., Zhong Z. (2021). Actively targeted nanomedicines for precision cancer therapy: Concept, construction, challenges and clinical translation. J. Control. Release.

[4] Tang L., Li J., Zhao Q., Pan T., Zhong H., Wang W. (2021). Advanced and Innovative Nano-Systems for Anticancer Targeted Drug Delivery. Pharmaceutics.

[5] Dadwal A., Baldi A., Narang R.K. (2018). Nanoparticles as carriers for drug delivery in cancer. Artif. Cells Nanomed. Biotechnol..

[6] Pearce A.K., O’Reilly R.K. (2019). Insights into active targeting of nanoparticles in drug delivery: Advances in clinical studies and design considerations for cancer nanomedicine. Bioconjug. Chem..

[7] Hejmady S., Pradhan R., Alexander A., Agrawal M., Singhvi G., Gorain B., Tiwari S., Kesharwani P., Dubey S.K. (2020). Recent advances in targeted nanomedicine as promising antitumor therapeutics. Drug Discov. Today.

[8] Zielińska A., Szalata M., Gorczyński A., Karczewski J., Eder P., Severino P., Cabeda J., Souto E., Słomski R. (2021). Cancer nanopharmaceuticals: Physicochemical characterization and in vitro/in vivo applications. Cancers.

[9] Molina-Crespo A., Cadete A., Sarrio D., Gamez-Chiachio M., Martinez L., Chao K., Olivera A., Gonella A., Diaz E., Palacios J. (2019). Intracellular delivery of an antibody targeting gasdermin-B Reduces HER2 breast cancer aggressiveness. Clin. Cancer Res..

[10] Ragelle H., Danhier F., Préat V., Langer R., Anderson D.G. (2016). Nanoparticle-based drug delivery systems: A commercial and regulatory outlook as the field matures. Expert Opin. Drug Deliv..

[11] Attia M.F., Anton N., Wallyn J., Omran Z., Vandamme T.F. (2019). An overview of active and passive targeting strategies to improve the nanocarriers efficiency to tumour sites. J. Pharm. Pharmacol..

[12] Rosenblum D., Joshi N., Tao W., Karp J.M., Peer D. (2018). Progress and challenges towards targeted delivery of cancer therapeutics. Nat. Commun..

[13] Nichols J.W., Bae Y.H. (2014). EPR: Evidence and fallacy. J. Control. Release.

[14] Raj S., Khurana S., Choudhari R., Kesari K.K., Kamal M.A., Garg N., Ruokolainen J., Das B.C., Kumar D. (2019). Specific targeting cancer cells with nanoparticles and drug delivery in cancer therapy. Semin. Cancer Biol..

[15] Heath V.L., Bicknell R. (2009). Anticancer strategies involving the vasculature. Nat. Rev. Clin. Oncol..

[16] Byrne J.D., Betancourt T., Brannon-Peppas L. (2008). Active targeting schemes for nanoparticle systems in cancer therapeutics. Adv. Drug Deliv. Rev..

[17] Bazak R., Houri M., El Achy S., Kamel S., Refaat T. (2014). Cancer active targeting by nanoparticles: A comprehensive review of literature. J. Cancer Res. Clin. Oncol..

[18] Muhamad N., Plengsuriyakarn T., Na-Bangchang K. (2018). Application of active targeting nanoparticle delivery system for chemotherapeutic drugs and traditional/herbal medicines in cancer therapy: A systematic review. Int. J. Nanomed..

[19] Autio K.A., Dreicer R., Anderson J., Garcia J.A., Alva A., Hart L.L., Milowsky M.I., Posadas E.M., Ryan C.J., Graf R.P. (2018). Safety and efficacy of bind-014, a docetaxel nanoparticle targeting prostate-specific membrane antigen for patients with metastatic castration-resistant prostate cancer. JAMA Oncol..

[20] Bazak R., Houri M., El Achy S., Hussein W., Refaat T. (2014). Passive targeting of nanoparticles to cancer: A comprehensive review of the literature. Mol. Clin. Oncol..

[21] Akhter H., Beg S., Tarique M., Malik A., Afaq S., Choudhry H., Hosawi S. (2020). Receptor-based targeting of engineered nanocarrier against solid tumors: Recent progress and challenges ahead. Biochim. et Biophys. Acta BBA-Gen. Subj..

[22] Akhtar M., Ahamed M., Alhadlaq H., Alrokayan S., Kumar S. (2014). Targeted anticancer therapy: Overexpressed receptors and nanotechnology. Clin. Chim. Acta.

[23] Zhu Y., Choi S.H., Shah K. (2015). Multifunctional receptor-targeting antibodies for cancer therapy. Lancet Oncol..

[24] Ahmad E., Ali A., Fatima M.T., Nimisha, Apurva, Kumar A., Sumi M.P., Sattar R.S.A., Mahajan B., Saluja S.S. (2021). Ligand decorated biodegradable nanomedicine in the treatment of cancer. Pharmacol. Res..

[25] Sonju J.J., Dahal A., Singh S.S., Jois S.D. (2020). Peptide-functionalized liposomes as therapeutic and diagnostic tools for cancer treatment. J. Control. Release.

[26] Kobayashi T., Ishida T., Okada Y., Ise S., Harashima H., Kiwada H. (2007). Effect of transferrin receptor-targeted liposomal doxorubicin in P-glycoprotein-mediated drug resistant tumor cells. Int. J. Pharm..

[27] Lei Z., Mengying Z., Yifei G., Xiangtao W., Meihua H. (2019). Alendronate-modified polydopamine-coated paclitaxel nanoparticles for osteosarcoma-targeted therapy. J. Drug Deliv. Sci. Technol..

[28] Gao X., Li L., Cai X., Huang Q., Xiao J., Cheng Y. (2020). Targeting nanoparticles for diagnosis and therapy of bone tumors: Opportunities and challenges. Biomaterials.

[29] Korc M., Chandrasekar B., Yamanaka Y., Friess H., Buchier M., Beger H.G. (1992). Overexpression of the epidermal growth factor receptor in human pancreatic cancer is associated with concomitant increases in the levels of epidermal growth factor and transforming growth factor alpha. J. Clin. Investig..

[30] Zimmermann M., Zouhair A., Azria D., Ozsahin M. (2006). The epidermal growth factor receptor (EGFR) in head and neck cancer: Its role and treatment implications. Radiat. Oncol..

[31] Minner S., Rump D., Tennstedt P., Simon R., Burandt E., Terracciano L., Moch H., Wilczak W., Bokemeyer C., Fisch M. (2011). Epidermal growth factor receptor protein expression and genomic alterations in renal cell carcinoma. Cancer.

[32] Herbst R.S., Shin D.M. (2002). Monoclonal antibodies to target epidermal growth factor receptor-positive tumors. Cancer.

[33] Senbanjo L.T., Chellaiah M.A. (2017). CD44: A Multifunctional Cell Surface Adhesion Receptor Is a Regulator of Progression and Metastasis of Cancer Cells. Front. Cell Dev. Biol..

[34] Yang C., Sheng Y., Shi X., Liu Y., He Y., Du Y., Zhang G., Gao F. (2020). CD44/HA signaling mediates acquired resistance to a PI3Kα inhibitor. Cell Death Dis..

[35] Ledermann J.A., Canevari S., Thigpen T. (2015). Targeting the folate receptor: Diagnostic and therapeutic approaches to personalize cancer treatments. Ann. Oncol..

[36] Zhao R., Diop-Bove N., Visentin M., Goldman I.D. (2011). Mechanisms of membrane transport of folates into cells and across epithelia. Annu. Rev. Nutr..

[37] Ghosh A., Heston W.D. (2004). Tumor target prostate specific membrane antigen (PSMA) and its regulation in prostate cancer. J. Cell. Biochem..

[38] Lütje S., Slavik R., Fendler W., Herrmann K., Eiber M. (2017). PSMA ligands in prostate cancer—Probe optimization and theranostic applications. Methods.

[39] Saniee F., Ravari N.S., Goodarzi N., Amini M., Atyabi F., Moghadam E.S., Dinarvand R. (2021). Glutamate-urea-based PSMA-targeted PLGA nanoparticles for prostate cancer delivery of docetaxel. Pharm. Dev. Technol..

[40] Ponka P., Lok C.N. (1999). The transferrin receptor: Role in health and disease. Int. J. Biochem. Cell Biol..

[41] Daniels T.R., Delgado T., Rodriguez J.A., Helguera G., Penichet M.L. (2006). The transferrin receptor part I: Biology and targeting with cytotoxic antibodies for the treatment of cancer. Clin. Immunol..

[42] Saxena M., Delgado Y., Sharma R.K., Sharma S., Guzmán S.L.P.D.L., Tinoco A.D., Griebenow K. (2018). Inducing cell death in vitro in cancer cells by targeted delivery of cytochrome c via a transferrin conjugate. PLoS ONE.

[43] Candelaria P.V., Leoh L.S., Penichet M.L., Daniels-Wells T.R. (2021). Antibodies targeting the transferrin receptor 1 (TfR1) as direct anti-cancer agents. Front. Immunol..

[44] Deshpande P., Jhaveri A., Pattni B., Biswas S., Torchilin V. (2018). Transferrin and octaarginine modified dual-functional liposomes with improved cancer cell targeting and enhanced intracellular delivery for the treatment of ovarian cancer. Drug Deliv..

[45] Soe Z.C., Kwon J.B., Thapa R.K., Ou W., Nguyen H.T., Gautam M., Oh K.T., Choi H.-G., Ku S.K., Yong C.S. (2019). Transferrin-conjugated polymeric nanoparticle for receptor-mediated delivery of doxorubicin in doxorubicin-resistant breast cancer cells. Pharmaceutics.

[46] De Freitas C.F., Montanha M.C., Pellosi D.S., Kimura E., Caetano W., Hioka N. (2019). Biotin-targeted mixed liposomes: A smart strategy for selective release of a photosensitizer agent in cancer cells. Mater. Sci. Eng. C.

[47] Ren W.X., Han J., Uhm S., Jang Y.J., Kang C., Kim J.-H. (2015). Recent development of biotin conjugation in biological imaging, sensing, and target delivery. Chem. Commun..

[48] Wang X., Zhang Q., Lv L., Fu J., Jiang Y., Xin H., Yao Q. (2017). Glioma and microenvironment dual targeted nanocarrier for improved antiglioblastoma efficacy. Drug Deliv..

[49] Wang S., Reinhard S., Li C., Qian M., Jiang H., Du Y., Lächelt U., Lu W., Wagner E., Huang R. (2017). Antitumoral cascade-targeting ligand for il-6 receptor-mediated gene delivery to glioma. Mol. Ther..

[50] Malaguarnera R., Belfiore A. (2011). The Insulin Receptor: A new target for cancer therapy. Front. Endocrinol..

[51] Nicolas J., Mura S., Brambilla D., Mackiewicz N., Couvreur P. (2013). Design, functionalization strategies and biomedical applications of targeted biodegradable/biocompatible polymer-based nanocarriers for drug delivery. Chem. Soc. Rev..

[52] Mazzotta E., De Benedittis S., Qualtieri A., Muzzalupo R. (2019). Actively targeted and redox responsive delivery of anticancer drug by chitosan nanoparticles. Pharmaceutics.

[53] Matini T., Francini N., Battocchio A., Spain S., Mantovani G., Vicent M.J., Sanchis J., Gallon E., Mastrotto F., Salmaso S. (2014). Synthesis and characterization of variable conformation pH responsive block co-polymers for nucleic acid delivery and targeted cell entry. Polym. Chem..

[54] Gallon E., Matini T., Sasso L., Mantovani G., De Benito A.A., Sanchis J., Caliceti P., Alexander C., Vicent M.J., Salmaso S. (2015). Triblock copolymer nanovesicles for pH-responsive targeted delivery and controlled release of siRNA to cancer cells. Biomacromolecules.

[55] Curcio M., Paolì A., Cirillo G., Di Pietro S., Forestiero M., Giordano F., Mauro L., Amantea D., Di Bussolo V., Nicoletta F. (2021). Combining dextran conjugates with stimuli-responsive and folate-targeting activity: A new class of multifunctional nanoparticles for cancer therapy. Nanomaterials.

[56] Venuta A., Moret F., Poggetto G.D., Esposito D., Fraix A., Avitabile C., Ungaro F., Malinconico M., Sortino S., Romanelli A. (2018). Shedding light on surface exposition of poly(ethylene glycol) and folate targeting units on nanoparticles of poly(ε-caprolactone) diblock copolymers: Beyond a paradigm. Eur. J. Pharm. Sci..

[57] Conte C., Fotticchia I., Tirino P., Moret F., Pagano B., Gref R., Ungaro F., Reddi E., Giancola C., Quaglia F. (2016). Cyclodextrin-assisted assembly of PEGylated polyester nanoparticles decorated with folate. Colloids Surf. B Biointerfaces.

[58] Poggetto G.D., Troise S.S., Conte C., Marchetti R., Moret F., Iadonisi A., Silipo A., Lanzetta R., Malinconico M., Quaglia F. (2020). Nanoparticles decorated with folate based on a site-selective αCD-rotaxanated PEG-b-PCL copolymer for targeted cancer therapy. Polym. Chem..

[59] Chiesa E., Dorati R., Conti B., Modena T., Cova E., Meloni F., Genta I. (2018). Hyaluronic acid-decorated chitosan nanoparticles for CD44-targeted delivery of everolimus. Int. J. Mol. Sci..

[60] Chiesa E., Riva F., Dorati R., Greco A., Ricci S., Pisani S., Patrini M., Modena T., Conti B., Genta I. (2020). On-Chip synthesis of hyaluronic acid-based nanoparticles for selective inhibition of CD44+ human mesenchymal stem cell proliferation. Pharmaceutics.

[61] Curcio M., Diaz-Gomez L., Cirillo G., Nicoletta F., Leggio A., Iemma F. (2021). Dual-targeted hyaluronic acid/albumin micelle-like nanoparticles for the vectorization of doxorubicin. Pharmaceutics.

[62] Cosco D., Mare R., Paolino D., Salvatici M.C., Cilurzo F., Fresta M. (2019). Sclareol-loaded hyaluronan-coated PLGA nanoparticles: Physico-chemical properties and in vitro anticancer features. Int. J. Biol. Macromol..

[63] Maiolino S., Russo A., Pagliara V., Conte C., Ungaro F., Russo G., Quaglia F. (2015). Biodegradable nanoparticles sequentially decorated with Polyethyleneimine and Hyaluronan for the targeted delivery of docetaxel to airway cancer cells. J. Nanobiotechnol..

[64] Russo A., Maiolino S., Pagliara V., Ungaro F., Tatangelo F., Leone A., Scalia G., Budillon A., Quaglia F., Russo G. (2016). Enhancement of 5-FU sensitivity by the proapoptotic rpL3 gene in p53 null colon cancer cells through combined polymer nanoparticles. Oncotarget.

[65] Gaio E., Conte C., Esposito D., Miotto G., Quaglia F., Moret F., Reddi E. (2018). Co-delivery of docetaxel and disulfonate tetraphenyl chlorin in one nanoparticle produces strong synergism between chemo- and photodynamic therapy in drug-sensitive and -resistant cancer cells. Mol. Pharm..

[66] Gaio E., Conte C., Esposito D., Reddi E., Quaglia F., Moret F. (2020). CD44 targeting mediated by polymeric nanoparticles and combination of chlorine TPCS2a-PDT and docetaxel-chemotherapy for efficient killing of breast differentiated and stem cancer cells in vitro. Cancers.

[67] Maiolino S., Moret F., Conte C., Fraix A., Tirino P., Ungaro F., Sortino S., Reddi E., Quaglia F. (2015). Hyaluronan-decorated polymer nanoparticles targeting the CD44 receptor for the combined photo/chemo-therapy of cancer. Nanoscale.

[68] Russo A., Pellosi D., Pagliara V., Milone M.R., Pucci B., Caetano W., Hioka N., Budillon A., Ungaro F., Russo G. (2016). Biotin-targeted Pluronic ^®^ P123/F127 mixed micelles delivering niclosamide: A repositioning strategy to treat drug-resistant lung cancer cells. Int. J. Pharm..

[69] Pellosi D.S., Calori I.R., De Paula L.B., Hioka N., Quaglia F., Tedesco A.C. (2017). Multifunctional theranostic Pluronic mixed micelles improve targeted photoactivity of Verteporfin in cancer cells. Mater. Sci. Eng. C.

[70] Conte C., Moret F., Esposito D., Poggetto G.D., Avitabile C., Ungaro F., Romanelli A., Laurienzo P., Reddi E., Quaglia F. (2019). Biodegradable nanoparticles exposing a short anti-FLT1 peptide as antiangiogenic platform to complement docetaxel anticancer activity. Mater. Sci. Eng. C.

[71] Voltan R., Secchiero P., Ruozi B., Forni F., Agostinis C., Caruso L., Vandelli M.A., Zauli G. (2013). nanoparticles engineered with rituximab and loaded with nutlin-3 show promising therapeutic activity in b-leukemic xenografts. Clin. Cancer Res..

[72] Belletti D., Tosi G., Riva G., Lagreca I., Galliania M., Luppi M., Vandelli M., Forni F., Ruozi B. (2015). Nutlin-3 loaded nanocarriers: Preparation, characterization and in vitro antineoplastic effect against primary effusion lymphoma. Int. J. Pharm..

[73] Akl M.A., Kartal-Hodzic A., Suutari T., Oksanen T., Montagner I.M., Rosato A., Ismael H.R., Afouna M.I., Caliceti P., Yliperttula M. (2019). Real-time label-free targeting assessment and in vitro characterization of curcumin-loaded poly-lactic-co-glycolic acid nanoparticles for oral colon targeting. ACS Omega.

[74] Colzani B., Speranza G., Dorati R., Conti B., Modena T., Bruni G., Zagato E., Vermeulen L., Dakwar G.R., Braeckmans K. (2016). Design of smart GE11-PLGA/PEG-PLGA blend nanoparticulate platforms for parenteral administration of hydrophilic macromolecular drugs: Synthesis, preparation and in vitro/ex vivo characterization. Int. J. Pharm..

[75] Laquintana V., Denora N., Lopalco A., Lopedota A., Cutrignelli A., Lasorsa F., Agostino G., Franco M. (2014). translocator protein ligand–plga conjugated nanoparticles for 5-fluorouracil delivery to glioma cancer cells. Mol. Pharm..

[76] Denora N., Laquintana V., Lopalco A., Iacobazzi R.M., Lopedota A., Cutrignelli A., Iacobellis G., Annese C., Cascione M., Leporatti S. (2013). In vitro targeting and imaging the translocator protein TSPO 18-kDa through G(4)-PAMAM–FITC labeled dendrimer. J. Control. Release.

[77] Iacobazzi R.M., Porcelli L., Lopedota A.A., Laquintana V., Lopalco A., Cutrignelli A., Altamura E., Di Fonte R., Azzariti A., Franco M. (2017). Targeting human liver cancer cells with lactobionic acid-G(4)-PAMAM-FITC sorafenib loaded dendrimers. Int. J. Pharm..

[78] Salerno M., Cenni E., Fotia C., Avnet S., Granchi D., Castelli F., Micieli D., Pignatello R., Capulli M., Rucci N. (2010). Bone-targeted doxorubicin-loaded nanoparticles as a tool for the treatment of skeletal metastases. Curr. Cancer Drug Targets.

[79] Cenni E., Avnet S., Granchi D., Fotia C., Salerno M., Micieli D., Sarpietro M.G., Pignatello R., Castelli F., Baldini N. (2012). The effect of poly(d,l-lactide-co-glycolide)-alendronate conjugate nanoparticles on human osteoclast precursors. J. Biomater. Sci. Polym. Ed..

[80] Clementi C., Miller K., Mero A., Satchi-Fainaro R., Pasut G. (2011). Dendritic Poly(ethylene glycol) Bearing Paclitaxel and Alendronate for Targeting Bone Neoplasms. Mol. Pharm..

[81] Cavalli R., Soster M., Argenziano M. (2016). Nanobubbles: A promising efficient tool for therapeutic delivery. Ther. Deliv..

[82] Jin Z., Chang J., Dou P., Jin S., Jiao M., Tang H., Jiang W., Ren W., Zheng S. (2020). Tumor targeted multifunctional magnetic nanobubbles for MR/US dual imaging and focused ultrasound triggered drug delivery. Front. Bioeng. Biotechnol..

[83] Xiao R., Zhao Z., Chen J., He L., Wang H., Huang L., Luo B. (2020). Preparation and ultrasonic imaging investigation of perfluoropentane-filled polylactic acid nanobubbles as a novel targeted ultrasound contrast agent. Front. Mater..

[84] Xu J.S., Huang J., Qin R., Hinkle G.H., Povoski S.P., Martin E.W., Xu R.X. (2010). Synthesizing and binding dual-mode poly (lactic-co-glycolic acid) (PLGA) nanobubbles for cancer targeting and imaging. Biomaterials.

[85] Zhang X., Zheng Y., Wang Z., Huang S., Chen Y., Jiang W., Zhang H., Ding M., Li Q., Xiao X. (2014). Methotrexate-loaded PLGA nanobubbles for ultrasound imaging and synergistic targeted therapy of residual tumor during HIFU ablation. Biomaterials.

[86] Capolla S., Argenziano M., Bozzer S., D’Agaro T., Bittolo T., De Leo L., Not T., Busato D., Dal Bo M., Toffoli G. (2021). Targeted chitosan nanobubbles as a new delivery approach required for anti-microRNA-17 based systemic therapy in Burkitt lymphoma models. Small.

[87] Argenziano M., Occhipinti S., Guiot C., Giovarelli M., Cavalli R. Nanobubble-based HER2 immunotherapy through dendritic cells targeting. Presented at the CRS Italy Chapter Workshop 2017.

[88] Gulati M., Grover M., Singh S., Singh M. (1998). Lipophilic drug derivatives in liposomes. Int. J. Pharm..

[89] Crommelin D.J., Van Hoogevest P., Storm G. (2019). The role of liposomes in clinical nanomedicine development. What now? Now what?. J. Control. Release.

[90] Shah S., Dhawan V., Holm R., Nagarsenker M.S., Perrie Y. (2020). Liposomes: Advancements and innovation in the manufacturing process. Adv. Drug Deliv. Rev..

[91] D’Avanzo N., Torrieri G., Figueiredo P., Celia C., Paolino D., Correia A., Moslova K., Teesalu T., Fresta M., Santos H.A. (2021). LinTT1 peptide-functionalized liposomes for targeted breast cancer therapy. Int. J. Pharm..

[92] Corti A., Curnis F., Rossoni G., Marcucci F., Gregorc V. (2013). peptide-mediated targeting of cytokines to tumor vasculature: The NGR-hTNF Example. BioDrugs.

[93] Zuccari G., Milelli A., Pastorino F., Loi M., Petretto A., Parise A., Marchetti C., Minarini A., Cilli M., Emionite L. (2015). Tumor vascular targeted liposomal-bortezomib minimizes side effects and increases therapeutic activity in human neuroblastoma. J. Control. Release.

[94] Di Paolo D., Pastorino F., Zuccari G., Caffa I., Loi M., Marimpietri D., Brignole C., Perri P., Cilli M., Nico B. (2013). Enhanced anti-tumor and anti-angiogenic efficacy of a novel liposomal fenretinide on human neuroblastoma. J. Control. Release.

[95] Morelli G., Accardo A., Tesauro D., Cicala C., Salzano G., De Rosa G., Morisco A., Aloj L., Aurilio M., Maione F. (2012). Peptide-modified liposomes for selective targeting of bombesin receptors overexpressed by cancer cells: A potential theranostic agent. Int. J. Nanomed..

[96] Accardo A., Mansi R., Salzano G., Morisco A., Aurilio M., Parisi A., Maione F., Cicala C., Ziaco B., Tesauro D. (2012). Bombesin peptide antagonist for target-selective delivery of liposomal doxorubicin on cancer cells. J. Drug Target..

[97] Burger J.A., Kipps T.J. (2006). CXCR4: A key receptor in the crosstalk between tumor cells and their microenvironment. Blood.

[98] Ierano’ C., Portella L., Lusa S., Salzano G., D’Alterio C., Napolitano M., Buoncervello M., Macchia D., Spada M., Barbieri A. (2016). CXCR4-antagonist Peptide R-liposomes for combined therapy against lung metastasis. Nanoscale.

[99] Ong K.L., Lam K.S.L., Cheung B.M.Y. (2005). Urotensin II: Its function in health and its role in disease. Cardiovasc. Drugs Ther..

[100] Zappavigna S., Abate M., Cossu A.M., Lusa S., Campani V., Scotti L., Luce A., Yousif A.M., Merlino F., Grieco P. (2019). Urotensin-II-targeted liposomes as a new drug delivery system towards prostate and colon cancer cells. J. Oncol..

[101] Rinaldi F., Hanieh P.N., Del Favero E., Rondelli V., Brocca P., Pereira M.C., Andreev O.A., Reshetnyak Y.K., Marianecci C., Carafa M. (2018). Decoration of Nanovesicles with pH (Low) Insertion Peptide (pHLIP) for Targeted Delivery. Nanoscale Res. Lett..

[102] Pereira M.C., Pianella M., Wei D., Moshnikova A., Marianecci C., Carafa M., Andreev O.A., Reshetnyak Y.K. (2016). pH-sensitive pHLIP^®^ coated niosomes. Mol. Membr. Biol..

[103] Uchegbu I.F., Vyas S.P. (1998). Non-ionic surfactant based vesicles (niosomes) in drug delivery. Int. J. Pharm..

[104] Lasic D. (1990). On the thermodynamic stability of liposomes. J. Colloid Interface Sci..

[105] Handjani-Vila R.M., Ribier A., Rondot B., Vanlerberghie G. (1979). Dispersions of lamellar phases of non-ionic lipids in cosmetic products. Int. J. Cosmet. Sci..

[106] Florence A.T. (1993). New drug delivery systems. Chem. Ind..

[107] Yeo P.L., Lim C.L., Chye S.M., Ling A.P.K., Koh R.Y. (2017). Niosomes: A review of their structure, properties, methods of preparation, and medical applications. Asian Biomed..

[108] Kuotsu K., Karim K.M., Mandal A.S., Biswas N., Guha A., Chatterjee S., Behera M. (2010). Niosome: A future of targeted drug delivery systems. J. Adv. Pharm. Technol. Res..

[109] Pozza E.D., Lerda C., Costanzo C., Donadelli M., Dando I., Zoratti E., Scupoli M., Beghelli S., Scarpa A., Fattal E. (2013). Targeting gemcitabine containing liposomes to CD44 expressing pancreatic adenocarcinoma cells causes an increase in the antitumoral activity. Biochim. et Biophys. Acta (BBA) Biomembr..

[110] Arpicco S., Lerda C., Pozza E.D., Costanzo C., Tsapis N., Stella B., Donadelli M., Dando I., Fattal E., Cattel L. (2013). Hyaluronic acid-coated liposomes for active targeting of gemcitabine. Eur. J. Pharm. Biopharm..

[111] Marengo A., Forciniti S., Dando I., Pozza E.D., Stella B., Tsapis N., Yagoubi N., Fanelli G., Fattal E., Heeschen C. (2018). Pancreatic cancer stem cell proliferation is strongly inhibited by diethyldithiocarbamate-copper complex loaded into hyaluronic acid decorated liposomes. Biochim. et Biophys. Acta (BBA)-Gen. Subj..

[112] Gazzano E., Buondonno I., Marengo A., Rolando B., Chegaev K., Kopecka J., Saponara S., Sorge M., Hattinger C.M., Gasco A. (2019). Hyaluronated liposomes containing H2S-releasing doxorubicin are effective against P-glycoprotein-positive/doxorubicin-resistant osteosarcoma cells and xenografts. Cancer Lett..

[113] Cano M.E., Lesur D., Bincoletto V., Gazzano E., Stella B., Riganti C., Arpicco S., Kovensky J. (2020). Synthesis of defined oligohyaluronates-decorated liposomes and interaction with lung cancer cells. Carbohydr. Polym..

[114] Santana-Armas M.L., De Ilarduya C.T. (2021). Strategies for cancer gene-delivery improvement by non-viral vectors. Int. J. Pharm..

[115] Ponti F., Campolungo M., Melchiori C., Bono N., Candiani G. (2021). Cationic lipids for gene delivery: Many players, one goal. Chem. Phys. Lipids.

[116] Surace C., Arpicco S., Dufaÿ-Wojcicki A., Marsaud V., Bouclier C., Clay D., Cattel L., Renoir J.-M., Fattal E. (2009). Lipoplexes targeting the CD44 hyaluronic acid receptor for efficient transfection of breast cancer cells. Mol. Pharm..

[117] Wojcicki A.D., Hillaireau H., Nascimento T.L., Arpicco S., Taverna M., Ribes S., Bourge M., Nicolas V., Bochot A., Vauthier C. (2012). Hyaluronic acid-bearing lipoplexes: Physico-chemical characterization and in vitro targeting of the CD44 receptor. J. Control. Release.

[118] Taetz S., Bochot A., Surace C., Arpicco S., Renoir J.-M., Schaefer U.F., Marsaud V., Kerdine-Roemer S., Lehr C.-M., Fattal E. (2009). Hyaluronic acid-modified DOTAP/dope liposomes for the targeted delivery of anti-telomerase siRNA to CD44-expressing lung cancer cells. Oligonucleotides.

[119] Nascimento T.L., Hillaireau H., Vergnaud J., Rivano M., Deloménie C., Courilleau D., Arpicco S., Suk J.S., Hanes J., Fattal E. (2016). Hyaluronic acid-conjugated lipoplexes for targeted delivery of siRNA in a murine metastatic lung cancer model. Int. J. Pharm..

[120] Nascimento T.L., Hillaireau H., Noiray M., Bourgaux C., Arpicco S., Pehau-Arnaudet G., Taverna M., Cosco D., Tsapis N., Fattal E. (2015). Supramolecular organization and siRNA binding of hyaluronic acid-coated lipoplexes for targeted delivery to the CD44 receptor. Langmuir.

[121] Leto I., Coronnello M., Righeschi C., Bergonzi M.C., Mini E., Bilia A.R. (2016). Enhanced efficacy of artemisinin loaded in transferrin-conjugated liposomes versus stealth liposomes against HCT-8 colon cancer cells. ChemMedChem.

[122] Salzano G., Marra M., Porru M., Zappavigna S., Abbruzzese A., La Rotonda M., Leonetti C., Caraglia M., De Rosa G. (2010). Self-assembly nanoparticles for the delivery of bisphosphonates into tumors. Int. J. Pharm..

[123] Ristori S., Grillo I., Lusa S., Thamm J., Valentino G., Campani V., Caraglia M., Steiniger F., Luciani P., De Rosa G. (2018). Structural characterization of self-assembling hybrid nanoparticles for bisphosphonate delivery in tumors. Mol. Pharm..

[124] Porru M., Zappavigna S., Salzano G., Luce A., Stoppacciaro A., Balestrieri Artuso S., Lusa S., De Rosa G., Leonetti C., Caraglia M. (2014). Medical treatment of orthotopic glioblastoma with transferrin-conjugated nanoparticles encapsulating zoledronic acid. Oncotarget.

[125] Salzano G., Zappavigna S., Luce A., D’Onofrio N., Balestrieri M.L., Grimaldi A., Lusa S., Ingrosso D., Artuso S., Porru M. (2016). Transferrin-targeted nanoparticles containing zoledronic acid as a potential tool to inhibit glioblastoma growth. J. Biomed. Nanotechnol..

[126] Scognamiglio I., Di Martino M.T., Campani V., Virgilio A., Galeone A., Gullà A., Cantafio M.E.G., Misso G., Tagliaferri P., Tassone P. (2014). Transferrin-Conjugated SNALPs Encapsulating 2′-O-Methylated miR-34a for the Treatment of Multiple Myeloma. BioMed Res. Int..

[127] Tavano L., Muzzalupo R., Mauro L., Pellegrino M., Andò S., Picci N. (2013). Transferrin-Conjugated Pluronic Niosomes as a New Drug Delivery System for Anticancer Therapy. Langmuir.

[128] Tavano L., Mauro L., Naimo G.D., Bruno L., Picci N., Andò S., Muzzalupo R. (2016). further evolution of multifunctional niosomes based on pluronic surfactant: Dual active targeting and drug combination properties. Langmuir.

[129] Paolino D., Licciardi M., Celia C., Giammona G., Fresta M., Cavallaro G. (2012). Folate-targeted supramolecular vesicular aggregates as a new frontier for effective anticancer treatment in in vivo model. Eur. J. Pharm. Biopharm..

[130] Scomparin A., Salmaso S., Eldar-Boock A., Ben-Shushan D., Ferber S., Tiram G., Shmeeda H., Landa-Rouben N., Leor J., Caliceti P. (2015). A comparative study of folate receptor-targeted doxorubicin delivery systems: Dosing regimens and therapeutic index. J. Control. Release.

[131] Gazzano E., Rolando B., Chegaev K., Salaroglio I.C., Kopecka J., Pedrini I., Saponara S., Sorge M., Buondonno I., Stella B. (2018). Folate-targeted liposomal nitrooxy-doxorubicin: An effective tool against P-glycoprotein-positive and folate receptor-positive tumors. J. Control. Release.

[132] Cheung N.K., Saarinen U.M., Neely J.E., Landmeier B., Donovan D., Coccia P.F. (1985). Monoclonal antibodies to a glycolipid antigen on human neuroblastoma cells. Cancer Res..

[133] Pagnan G., Montaldo P.G., Pastorino F., Raffaghello L., Kirchmeier M., Allen T.M., Ponzoni M. (1999). GD2-mediated melanoma cell targeting and cytotoxicity of liposome-entrapped fenretinide. Int. J. Cancer.

[134] Brown B.S., Patanam T., Mobli K., Celia C., Zage P.E., Bean A.J., Tasciotti E. (2014). Etoposide-loaded immunoliposomes as active targeting agents for GD2-positive malignancies. Cancer Biol. Ther..

[135] Scavo M.P., Cutrignelli A., DePalo N., Fanizza E., Laquintana V., Gasparini G., Giannelli G., Denora N. (2020). Effectiveness of a controlled 5-FU delivery based on FZD10 antibody-conjugated liposomes in colorectal cancer in vitro models. Pharmaceutics.

[136] Ruozi B., Riva G., Belletti D., Tosi G., Barozzi P., Luppi M., Forni F., Vandelli M.A. (2010). Immunoliposomal systems targeting primary effusion lymphoma: In vitro study. Nanomedicine.

[137] Belletti D., Vandelli M.A., Tonelli M., Zapparoli M., Forni F., Tosi G., Ruozi B. (2014). Functionalization of liposomes: Microscopical methods for preformulative screening. J. Liposome Res..

[138] Bragagni M., Mennini N., Ghelardini C., Mura P. (2012). Development and characterization of niosomal formulations of doxorubicin aimed at brain targeting. J. Pharm. Pharm. Sci..

[139] Barattin M., Mattarei A., Balasso A., Paradisi C., Cantù L., Del Favero E., Viitala T., Mastrotto F., Caliceti P., Salmaso S. (2018). pH-controlled liposomes for enhanced cell penetration in tumor environment. ACS Appl. Mater. Interfaces.

[140] Popilski H., Feinshtein V., Kleiman S., Mattarei A., Garofalo M., Salmaso S., Stepensky D. (2020). Doxorubicin liposomes cell penetration enhancement and its potential drawbacks for the tumor targeting efficiency. Int. J. Pharm..

[141] Iwamaru Y., Shimizu Y., Imamura M., Murayama Y., Endo R., Tagawa Y., Ushiki-Kaku Y., Takenouchi T., Kitani H., Mohri S. (2008). Lactoferrin induces cell surface retention of prion protein and inhibits prion accumulation. J. Neurochem..

[142] Pireddu R., Pibiri M., Valenti D., Sinico C., Fadda A.M., Simbula G., Lai F. (2018). A novel lactoferrin-modified stealth liposome for hepatoma-delivery of triiodothyronine. Int. J. Pharm..

[143] Gérard A.-C., Daumerie C., Mestdagh C., Gohy S., De Burbure C., Costagliola S., Miot F., Nollevaux M.-C., Denef J.-F., Rahier J. (2003). correlation between the loss of thyroglobulin iodination and the expression of thyroid-specific proteins involved in iodine metabolism in thyroid carcinomas. J. Clin. Endocrinol. Metab..

[144] Paolino D., Cosco D., Gaspari M., Celano M., Wolfram J., Voce P., Puxeddu E., Filetti S., Celia C., Ferrari M. (2014). Targeting the thyroid gland with thyroid-stimulating hormone (TSH)-nanoliposomes. Biomaterials.

[145] Duong V.-A., Nguyen T.-T., Maeng H.-J. (2020). Preparation of Solid lipid nanoparticles and nanostructured lipid carriers for drug delivery and the effects of preparation parameters of solvent injection method. Molecules.

[146] Magro R.D., Ornaghi F., Cambianica I., Beretta S., Re F., Musicanti C., Rigolio R., Donzelli E., Canta A.R., Ballarini E. (2017). ApoE-modified solid lipid nanoparticles: A feasible strategy to cross the blood-brain barrier. J. Control. Release.

[147] Béduneau A., Saulnier P., Benoit J.-P. (2007). Active targeting of brain tumors using nanocarriers. Biomaterials.

[148] Cortesi R., Menegatti E., Esposito E., Ravani L., Drechsler M. (2011). Colloidal dispersions for the delivery of acyclovir: A comparative study. Indian J. Pharm. Sci..

[149] Mohammadi-Samani S., Ghasemiyeh P. (2018). Solid lipid nanoparticles and nanostructured lipid carriers as novel drug delivery systems: Applications, advantages and disadvantages. Res. Pharm. Sci..

[150] Battaglia L., Muntoni E., Chirio D., Peira E., Annovazzi L., Schiffer D., Mellai M., Riganti C., Salaroglio I.C., Lanotte M. (2017). Solid lipid nanoparticles by coacervation loaded with a methotrexate prodrug: Preliminary study for glioma treatment. Nanomedicine.

[151] Muntoni E., Martina K., Marini E., Giorgis M., Lazzarato L., Salaroglio I.C., Riganti C., Lanotte M., Battaglia L. (2019). Methotrexate-loaded solid lipid nanoparticles: Protein functionalization to improve brain biodistribution. Pharmaceutics.

[152] Desmaële D., Gref R., Couvreur P. (2012). Squalenoylation: A generic platform for nanoparticular drug delivery. J. Control. Release.

[153] Valetti S., Maione F., Mura S., Stella B., Desmaële D., Noiray M., Vergnaud J., Vauthier C., Cattel L., Giraudo E. (2014). Peptide-functionalized nanoparticles for selective targeting of pancreatic tumor. J. Control. Release.

[154] Valetti S., Mura S., Noiray M., Arpicco S., Dosio F., Vergnaud J., Desmaële D., Stella B., Couvreur P. (2014). Peptide Conjugation: Before or After Nanoparticle Formation?. Bioconjug. Chem..

[155] Chirio D., Peira E., Sapino S., Chindamo G., Oliaro-Bosso S., Adinolfi S., Dianzani C., Baratta F., Gallarate M. (2021). A new bevacizumab carrier for intravitreal administration: Focus on stability. Pharmaceutics.

